# Targeting cancer via macrophage-derived exosomal miRNAs: implications for tumor progression and resistance

**DOI:** 10.3389/fimmu.2025.1683799

**Published:** 2025-11-20

**Authors:** Prasanna Srinivasan Ramalingam, Muhammad Afzal, M. Arockia Babu, Rekha M. M., Samir Sahoo, Surya Nath Pandey, Haider Ali, Md Sadique Hussain, Gaurav Gupta, Janaki Ramaiah Mekala, Sivakumar Arumugam

**Affiliations:** 1 Protein Engineering Lab, School of Biosciences and Technology, Vellore Institute of Technology, Vellore, Tamil Nadu, India; 2 Department of Pharmaceutical Sciences, Pharmacy Program, Batterjee Medical College, Jeddah, Saudi Arabia; 3 Institute of Pharmaceutical Research, GLA University, Mathura, Uttar Pradesh, India; 4 Department of Chemistry and Biochemistry, School of Sciences, JAIN (Deemed to Be University), Bengaluru, Karnataka, India; 5 Department of General Medicine, IMS & SUM Hospital, Siksha ‘O’ Anusandhan (Deemed to be University), Bhubaneswar, Odisha, India; 6 Department of Pharmacology, Teerthanker Mahaveer College of Pharmacy, Teerthanker Mahaveer University, Moradabad, Uttar Pradesh, India; 7 Department of Pharmacology, Kyrgyz State Medical College, Bishkek, Kyrgyzstan; 8 Uttaranchal Institute of Pharmaceutical Sciences, Uttaranchal University, Dehradun, Uttarakhand, India; 9 Centre for Research Impact and Outcome-Chitkara College of Pharmacy, Chitkara University, Rajpura, Punjab, India; 10 Centre of Medical and Bio-allied Health Sciences Research, Ajman University, Ajman, United Arab Emirates; 11 Department of Integrative Biology, School of Biosciences and Technology, Vellore Institute of Technology, Vellore, Tamil Nadu, India

**Keywords:** cancer biomarkers, exosomal miRNAs, macrophage-derived exosomes, therapeutic resistance, tumor-associated macrophages, tumor microenvironment

## Abstract

Recent studies on macrophages showed their contribution to tumorigenesis, progression, metastasis, and chemoresistance by influencing the local tumor microenvironment and cancer cells. Exosomes form a subset of extracellular vesicles and have played a major role in the interaction between cancer cells and macrophages. This review intends to discuss the existing literature on employing macrophage-derived exosomes as a vehicle for microRNA (miRNA) delivery in oncological applications. It will evaluate the molecular principles of this therapeutic approach and its capacity to enhance cancer therapy by elucidating problems like drug and radio-resistance. This review uniquely emphasizes the diagnostic and therapeutic potential of macrophage-derived exosomal miRNAs, summarizing current understandings into their molecular processes, tumor specificity, and strategies to overcome therapeutic resistance. This review synthesizes recent studies and evaluates how macrophage-derived exosomes and their miRNAs contribute to cancers. These vesicles are multipurpose tools that regulate tumor behavior, considering they can regulate it through post-transcriptional regulation and protein phosphorylation. Such exosomes that are engineered can potentially introduce a novel dimension because they have the capability of delivering targeted oncogenic or tumor-suppressive miRNAs to overcome limitations of current cancer therapeutics, particularly drug and radioresistance. Engineered macrophage-derived exosomes may thus have the potential as a novel approach for cancer treatment and overcoming therapeutic resistance.

## 1 Introduction

Exosomes are extracellular vesicles (EVs), typically 30–150 nm in diameter, secreted by various cells, such as cancer and immune cells or stromal cells (1). Proteins, nucleic acids, and lipids carried by these vesicles are biologically active and mediate intercellular communication (2). Exosomes have been in the limelight in the field of oncology and are already regarded as significant contributors to the tumor microenvironment (TME) (3). They participate in numerous processes essential to tumorigenesis, e.g., tumor growth, metastasis, immune evasion, and the development of drug resistance (4). The fact that exosomes can induce plasticity in the recipient cells, mostly through inciting malignant transformation, makes them an indispensable to cancer development and a target for therapeutic intervention (5, 6). Exosomes form as intraluminal vesicles (ILVs) in multivesicular bodies (MVBs) and are released by fusion with the plasma membrane; their proteins, lipids, and nucleic acid cargo can reprogram recipient cells within the TME (7). In cancers, tumor-associated macrophages (TAMs) tend to be biased towards immunoregulatory M2-like phenotypes, with the M1-like macrophages being pro-inflammatory. Meanwhile, macrophage-derived exosomes (MDEs) are exosomes that are released by macrophages; their microRNA (miRNA) cargo (MDE-miRNAs) can be suggestive of macrophage polarization and local stimuli and reorganize tumor growth, metastasis, immune evasion, and therapeutic resistance (8, 9). MDEs may play an important role in cancer biology (10). The TAMs are primarily polarized to a pro-tumorigenic phenotype (11). These TAMs will be involved in circuits maintaining tumor growth, angiogenesis, or invasion/metastasis, which may reprogram cancer behavior (12). By transmitting such a load, MDEs stimulate tumor cell survival and growth and invasion, and the development of an immunosuppressed microenvironment hostile to immune editing by tumor cells (13, 14).

Recent advancements in studies have navigated this avenue of MDE therapeutics, particularly with regard to the delivery of miRNA to cancer cells. Selective treatment based on the affinity of MDEs to areas of tumors creates an appealing treatment strategy (15). Targeted delivery of therapeutic miRNAs resulting in gene modulation via MDEs has the potential to prevent the advancement of cancer and overcome the limitations present in conventional nanocarriers (16). In addition, individual variability in MDEs across cancers is the rationale for personalized medicine approaches (17). MiRNA content personalization in MDEs with the help of the tumor environment signature can lead to amelioration and personalized intervention (18). Developments in our understanding of the complexity of MDEs in cancer hold great possibilities to be further exploited as potential markers and as therapeutic vehicles in this context to allow the creation of specific new treatment paradigms (19). Even though exosome biogenesis is common across cell types, it is the cell of origin that leaves a mark on cargo and cellular functions (20). Relative to tumor-derived exosomes (TDEs) that tend to exacerbate oncogenic messaging in cancer cells, MDEs predominantly inform the TME and remodel cancer cells, stromal cells, and immunity by miRNAs associated with the macrophage state. They also exhibit tropism for hypoxic tumor subregions, and MDEs carry immunomodulatory signals that are less noticeable in alternative stromal exosomes. Although there is growing evidence on the diverse functions, there is inadequate systematic knowledge on how macrophage-derived exosomal miRNAs determine tumor biology and respond to therapy. The review is a summary of contemporary information on their mechanistic contributions, clinical associations with multiple large-scale cancer types, and obstacles to utilizing them in translation research. Given their dual potential role in tumor promotion and suppression, these exosomes represent a promising yet underexplored therapeutic avenue. The primary focus in this review is MDEs; other exosomes released by tumor cells or other stromal cells are only mentioned in the form of contrast where necessary. Understanding their molecular mechanisms and translational potential could unlock innovative strategies to overcome drug and radioresistance, paving the way for more precise and effective cancer treatments. In this review, we have explored the potential of Macrophage-derived exosomal miRNAs in tumor progression and resistance.

## 2 Biogenesis of macrophage-derived exosomes

### 2.1 Mechanisms of exosome formation

**Figure 1:**
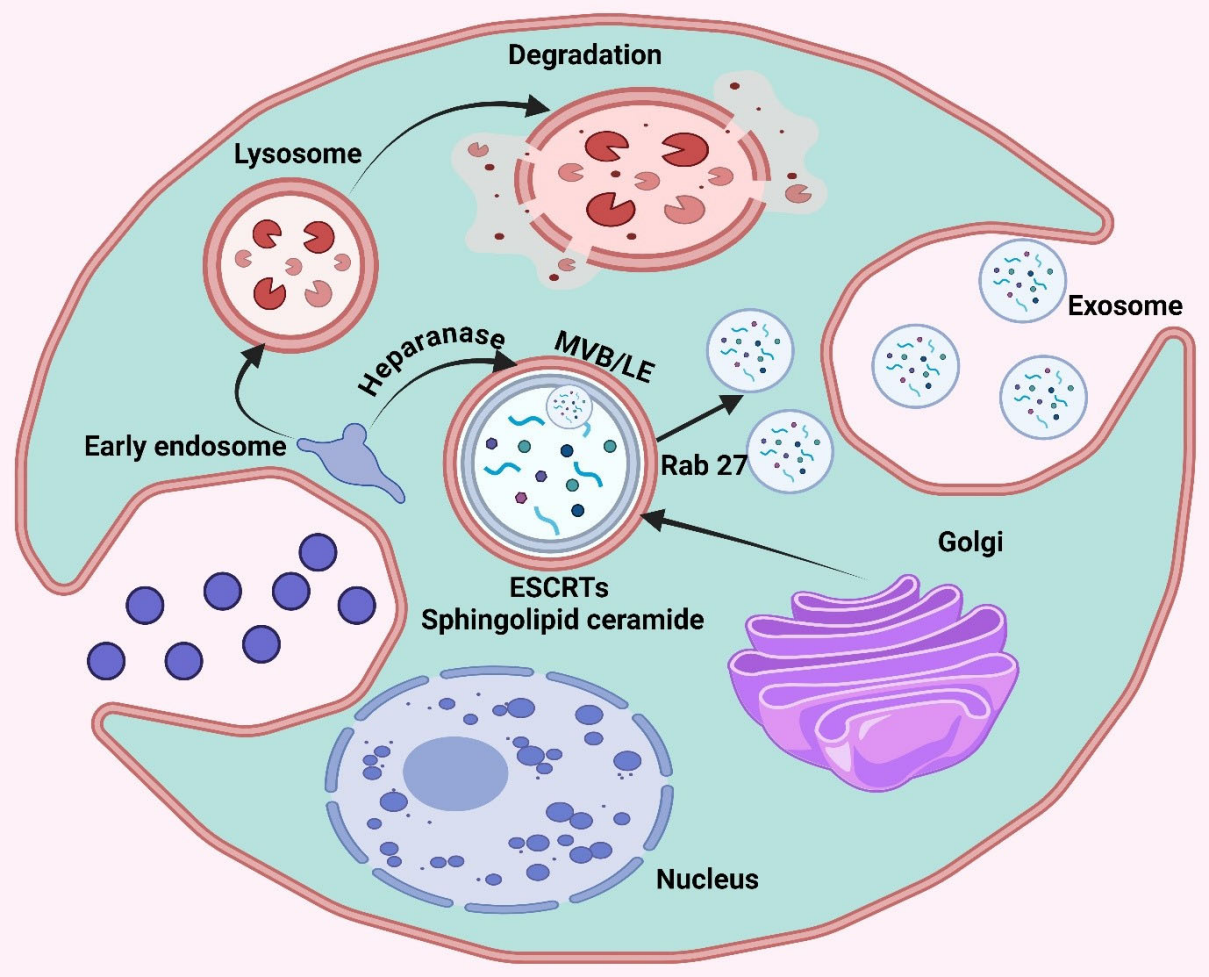
The graphic illustrates the cellular process of exosome production. This diagram depicts the process of early endosome creation leading to the production of multivesicular bodies (MVB/LE) and the eventual release of exosomes, as well as the routes involved in lysosomal degradation. Important compounds include ESCRT, Rab27, and sphingolipid ceramide.

The biogenesis of exosomes initiates with the internal budding of the cellular plasma membrane, leading to small vesicle-like structures called early endosomes; this is followed by a highly complex process known as MVBs formation (21). Subsequent invagination of these early endosomes leads to the formation of ILVs within MVBs (22). One of these steps is the formation of ILVs, which are important precursor vesicles containing bioactive cargo that will ultimately be delivered to recipient cells in exosomes (23). The membrane of these endosomes is then remodeled and organized into discrete domains that are enriched in particular proteins, lipids, or nucleic acids (24). This sorting process is mostly selective, by which only specific molecules are included in exosomes (25). After MVBs reach full maturation, they either fuse with lysosomes for degradation or fuse with the plasma membrane and release ILVs as exosomes into the extracellular space (26). The choice between these fates is controlled by several signaling pathways and molecule interactions that are the subject matter of ongoing research (27). At the heart of this endosomal maturation process are Ras-associated binding (Rab) GTPases that direct MVBs to fuse with the cell surface through SNARE protein-dependent final fusion (28). Exosomes are released into the extracellular space to act as a mode of intercellular communication, particularly within the TME, thus influencing cancer progression (**Figure 1**) (29). The polarization state (M1 vs. M2/TAM) of macrophages in particular alters miRNA abundance and loading in exosomes through established sorting pathways to remodel the exosomal repertoire.

Polarization leaves different miRNA signatures on the MDEs with functional implications in the tumor. M1-polarized macrophages specifically pack miR-155-5p into exosomes (greater in M1 than in M0/M2), and M1-exosomal miRNA can have anti-tumor effects in several models. Conversely, the reports of M2/TAM exosomes being repeatedly highly enriched with oncogenic miRNAs that engage cancer cells and trigger activated pro-survival signatures and invasion, and chemoresistance are recurrently mentioned (30). Mechanistically, such polarization-specific differences in cargo fold into general pathways of exosome sorting, hnRNPA2B1 attachment to EXOmotifs (31), YBX1-selection (e.g., of miR-223) (32), relative enrichment of 3′-uridylated isoforms in exosomes (33), and ceramide/neutral sphingomyelinase 2 (nSMase2)-mediated miRNA export (34) to provide a macrophage-specific basis to how M1 versus M2 states bias the exosomes-mediated miRNA positing.

### 2.2 Role of ESCRT machinery and other molecular players

The endosomal sorting complexes required for transport (ESCRT) machinery is an ATP-dependent mechanism that functions in the packaging of cargo into intraluminal vesicles (ILVs) within multivesicular bodies (MVBs), a critical step during exosome biogenesis (35). The ESCRT machine consists of four large complexes that include ESCRT-0, I, II, and III. Various complexes perform different roles in the process of exosome production (36). The major role of ESCRT-0 is to identify and enclose the cargo tagged with ubiquitin at the endosome membrane. During the formation of the MVBs, ILVs are generated by the process catalyzed by ESCRT-I and ESCRT-II. Meanwhile, ESCRT-III performs a last separation by breaking membranes to release nascent ILVs inside the lumen of MVB (37).

A wide variety of accessory proteins has also been shown to be involved in exosome biogenesis in addition to the ESCRT complexes (38). Alix and TSG101 are significant regulators that get involved with the ESCRT apparatus in orchestrating proper cargo sorting/packaging. Alix plays a critical role in the process of budding, and TSG101 associates with the ubiquitinated protein to mark them in the immature exosomes (39). Along with this, other molecular elements such as Rab GTPases are also necessary to release exosomes in the plasma membrane through transport and docking of MVBs (40). Two of the Rab family members, namely Rab27a and Rab27b have been demonstrated as being critical in the localization of MVBs near the plasma membrane through exosome release. Newer studies have also indicated the orchestration of the lipid nanodomains (also called as room rafts) in the exosome biogenesis (41). These membrane lipid-rich areas serve as protein complex scaffolds that participate in exosome formation (42). Consequently, the biophysical nature of such microdomains is significantly modulated by cholesterol, sphingomyelin, and ceramide, and hence various abilities to convert them to exosomes (43).

### 2.3 Influence of external stimuli

The generation and secretion of MDE are induced by external factors such as hypoxia, inflammation, or oxidative stress. EV content changes in the hypoxic, acidic, and low glucose TME, which leads to alterations in their function (44). Hypoxia is a hallmark of solid tumors known to promote the secretion of exosomes from cancer cells and TAMs (45). Exosomal cargo is likewise altered under hypoxic conditions that tend to enrich for pro-angiogenic factors such as miRNA and vascular endothelial growth factor (VEGF), enhancing metastasis. These observations suggest that not only does hypoxia produce exosomes, but it also modifies their cargo to the advantage of tumor progression (46). In line with its inflammatory status, which is well characterized in cancer, exosome biogenesis was also altered. The released exosomes contain additional protein and RNA cargo when macrophages are exposed to inflammatory cytokines such as TNF-α or IL-6 (47, 48). Studies have demonstrated that exosomes secreted from inflamed cells can deliver immunosuppressive molecules to dampen the activation of cytotoxic T cells and facilitate immune evasion (49). On the other hand, cellular stress, such as that induced by oxidative stress and nutrient deprivation or provoked by treatment with chemotherapeutic agents, may result in increased exosome secretion (50, 51). This stress-induced exosomal secretion is believed to represent a cytoprotective process that removes deleterious proteins and RNAs from cells, as well as modulates the TME, which further influences cancer cell proliferation/survival in response to treatment (52).

## 3 Functions of MDEs in cancer

The MDEs significantly contribute to tumor biology through intercellular communication in the TME. These vesicles convey regulatory miRNAs, proteins, and lipids that significantly affect cancer cells’ surrounding stroma. MDEs can lead to tumor progression, angiogenesis, invasion, and immune suppression and therapy resistance depending on their molecular cargo (53). The subsections below provide an overview of the key MDE-regulated processes in cancer progression.

### 3.1 MDEs promote tumor proliferation

One of the major functionalities that MDEs carry out in case of cancer is to create a suitable condition for tumor growth and proliferation (54). Oncogenic dysregulation of MDEs often involves oncogenic miRNAs and proteins to instigate signaling pathways in human cancer cells, e.g., PI3K/AKT or Wnt/β-catenin pathway that brings about increased cell proliferation/survival (55). MiR-501-3p in M2-exosomes favors tumor progression by triggering the transforming growth factor-β (TGF-β) cascade and suppressing the tumor suppressor gene TGFBR3 (56). In human epithelial ovarian cancer (EOC), exosomal miR-221−3p derived from M2 macrophages promotes cancer growth by reducing the cyclin-dependent kinase inhibitor 1B (CDKN1B) (57). A separate investigation indicates that exosomal miR-29a-3p and miR-21−5p derived from M2 macrophages increase the proportion of T regulatory cells (Tregs) to T helper cell 17 (Th17), thereby contributing to a tumor immune inhibitory TME and facilitating the development of cancer and metastasis (58). The data indicate that macrophage-derived exosomal miRNAs target not only cancerous cells directly but also the immune systems, thereby influencing cancer cells indirectly.

### 3.2 MDEs promote metastasis

Moreover, MDEs modulate the TME by modifying stromal cell behavior, favoring angiogenesis, and establishing a permissive niche for many phases of tumorigenesis. MDEs are also involved in promoting the metastatic ability of cancer cells (59, 60). Through the transmission of pro-metastatic molecules, MDEs can reshape extracellular matrices to promote cancer cell invasiveness and assist in developing pre-metastatic niches at target organs (61). M2-Exos carry lncRNA AFAP1-AS1, downregulating miR-26a and upregulating activating transcription factor 2 (ATF2), hence facilitating esophageal cancer (EC) penetration and metastasis. Engaging M2 macrophages and the lncRNA AFAP1AS1/miR-26a/ATF2 pathway is a promising treatment option for EC (62). Lan et al. observed that M2-Exos regulate Brahma-related gene 1 (BRG1) by delivering miR-21 and miR-155-5p, leading to the downregulation of BRG1 and promoting colorectal cancer (CRC) metastasis (63).

### 3.3 MDEs confer drug resistance

Furthermore, MDEs are also involved in drug resistance to cancer. They can take up and transmit miRNAs, as well as other molecules that regulate the expression of drug-resistance genes in cancer cells, which helps reduce both chemotherapeutic response and response to targeted therapies (64, 65). Such exosome-mediated communication develops a multidrug-resistant and aggressive tumor phenotype. Recent data indicate that MDEs-derived miRNAs exert an inhibitory effect on cancer. MDEs, rich in miR-7, are internalized by EOC cells following treatment with tumor necrosis factor-like weak inducer of apoptosis (TWEAK). This internalization inhibits cell invasion by targeting the EGFR-mediated AKT/ERK system (66). MDEs miR-let-7a-5p can be transferred to lung cancer cells, resulting in the inhibition of cell growth, migration, and invasion through the downregulation of Bcl2-like 1 (BCL2L1) expression (67).

### 3.4 MDEs regulate immune responses

MDEs partake in the immune regulation within the TME (68). Consequently, these cells can modulate anti-tumor immune responses by affecting the function of different types of innate and adaptive immunity effector cells, leading to immunostimulants and cancer progression (69).

## 4 miRNA loading and cargo selection in MDEs

MiRNA loading and cargo selection in MDEs is a tightly regulated transcriptional program that dictates response to various stimuli, including cancer (70). Besides, the selective incorporation of specific miRNAs and other bioactive molecules into MDEs is not random, as specific cellular mechanisms mediate this process to provide directed signaling to recipient cells. MiRNA incorporation into MDEs occurs during the biogenesis of these vesicles in parent mesenchymal stromal/stem cells (MSCs) (71, 72). Many important proteins and pathways participate in modulating the selective packaging of miRNAs (73, 74). Key players for recognizing and mediating the loading of selected miRNAs into MDEs are those represented by RNA-binding proteins (RBPs), such as Argonaute 2 (Ago2), heterogeneous nuclear ribonucleoproteins, and endosomal sorting complexes required for transport (75, 76). Exosome-targeting motifs (EXO-motifs; GGAG/UGGA-like motifs) and U/CA-rich elements are preferentially bound by hnRNP family proteins, in macrophages, enhancing their export in budding intraluminal vesicles. The miRNAs that interact with Ago2 are selectively targeted to exosomes by a combination of their stable seed imaginary pairing potential and their preference for 5’-nucleotides to interact with Ago2. Additional sequence-independent sorting bias modulates RBP affinity/retention, versus export decisions are affected by secondary structure and 3-end modifications. A combination of these features can be used to operationalize the selective, non-random packaging of miRNAs into macrophage-derived exosomes. In practice, these RBPs read the above EXO-motifs, U/CA-rich elements, 3′-end marks, and exposed hairpins to triage individual miRNAs for vesicular export, committing them as cargo precursors during ILV budding within maturing MVBs (77). The miRNAs are then loaded into the intraluminal vesicles, which, after the bulbs are fused to the plasma membrane, lead to the release of MDEs out of the cell. The miRNA content of MDEs may reflect the physiological status of MSCs and even stimuli from external surroundings (78, 79). Under hypoxic conditions or in response to inflammatory signals, MSCs may selectively furnish MDEs with different miRNA cargos (i.e., pro-angiogenic miRs for TME redirecting recipient cancer cells and immunomodulatory miRs preventing anti-tumoral immune cell activation) (80). Hypoxia, which is a characteristic of solid tumors, enhances exosome release by TAMs and cargo composition, in part by altering the expression and post-translational status of RBPs, favoring certain motifs and diminishing others (81). Cellular stress and inflammatory cytokines also reprogram exosomes’ biogenesis and cargo selection, enriching pro-angiogenic or immunomodulatory miRNAs and reconfiguring the TME (82). Such context-dependent modifications justify the reason why exosomes of hypoxia-related, macrophage-produced exosomes often carry pro-metastatic/therapy-resistance miRNAs, which are evident throughout Section 5.

In addition to miRNAs, MDEs also selectively incorporate other molecular cargo, such as proteins, lipids, and lncRNA, under specific cellular contexts to mediate a targeted biological effect (83–85). The cargo sorting is indispensable to the function of MDEs in regulating the TME, promoting metastasis, and inducing drug resistance (86). The interaction between miRNAs and other cargo components in MDEs may enhance their potency on recipient cells synergistically (87). The miRNA cargo of MDEs in cancer can downregulate tumor suppressor genes or upregulate oncogenes in recipient cells, leading to the promotion of tumorigenesis and metastasis. Additionally, MDEs could be artificially designed to carry tumor-suppressive miRNAs and serve as a therapeutic strategy (88–90). Elucidating these processes in MDEs will be critical for the future design of targeted therapies that either suppress the pathological functions of MDEs or use them to deliver therapeutic molecules in cancer and potentially other diseases (91).

## 5 Applications of miRNA-loaded macrophage-derived exosomes as delivery vehicles

For a cross-comparison of key MDE-miRNAs, their validated targets, and net functional outcome across cancers, refer to **Table 1**.

**Table 1:** Comparative summary of macrophage-derived exosomal (MDE) miRNAs across cancers.

Cancer type	Key MDE-miRNA(s)	Validated direct target(s) in recipient cell	Functional outcome	References
Ovarian (EOC)	miR-223	PTEN	Tumor-promoting (chemoresistance via PI3K/AKT activation).	(92)
Gastric	miR-21	PTEN	Tumor-promoting (cisplatin resistance; PI3K/AKT).	(93)
PDAC	miR-365	CDA induction/nucleotide pool remodeling (gemcitabine inactivation)	Tumor-promoting (drug resistance).	(94)
PDAC	miR-501-3p	TGFBR3	Tumor-promoting (TGF-β activation; invasion/metastasis).	(56)
NSCLC	miR-155; miR-196a-5p	RASSF4	Tumor-promoting (EMT, migration, metastasis).	(95)
CRC	miR-155-5p	ZC3H12B (↑IL-6 stability)	Tumor-promoting (immune escape/tumor formation).	(96)
Melanoma	miR-29c-3p (from M1 macrophages)	ENPP2	Suppressive (reduced aggressiveness).	(97)

### 5.1 Lung cancer

**Figure 2:**
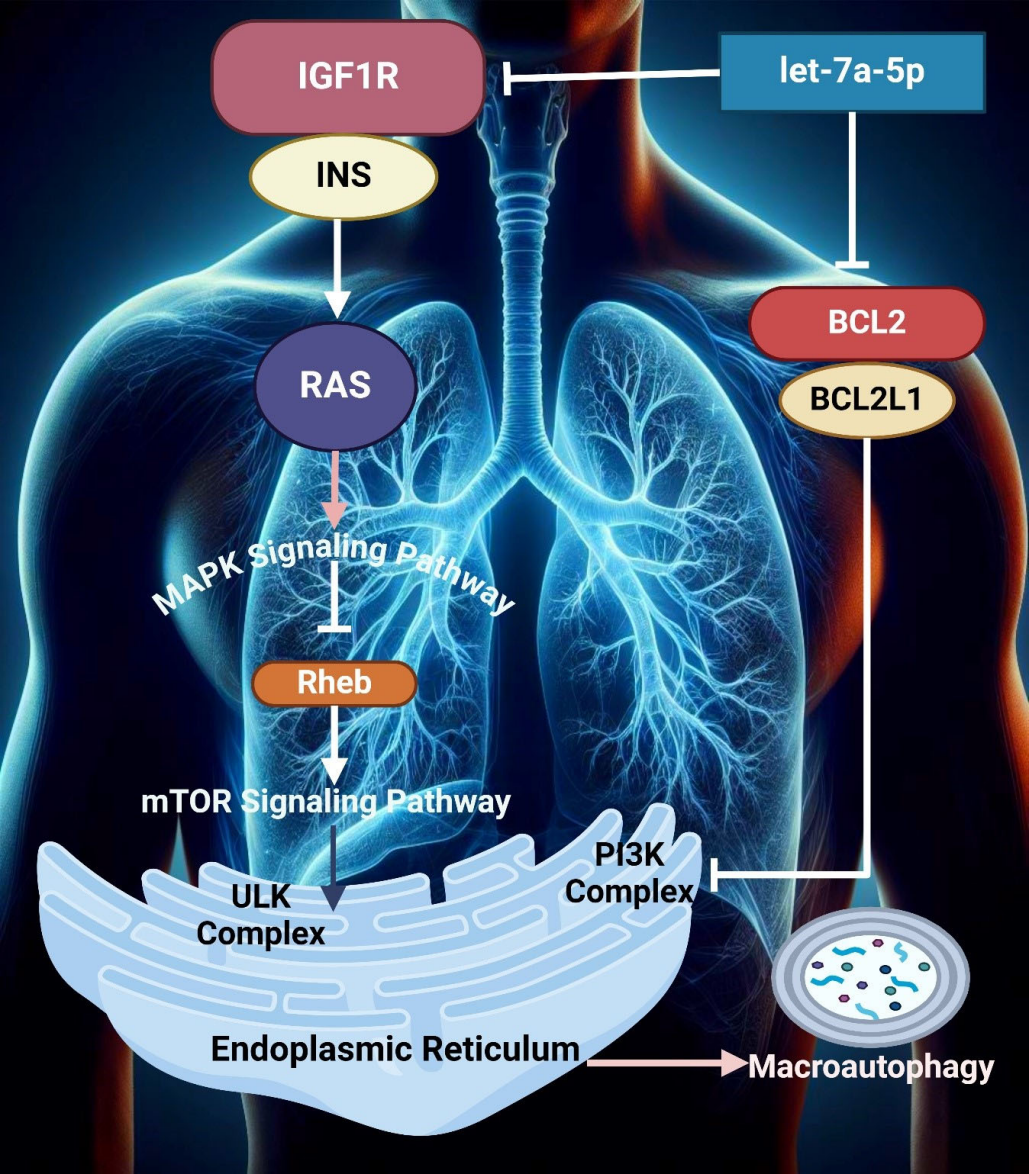
The image illustrates key signaling pathways involved in lung cellular processes, highlighting the MAPK and mTOR pathways. It depicts the interaction between IGF1R, INS, and RAS and the downstream effects on autophagy through the endoplasmic reticulum, mediated by Rheb and PI3K complexes.

Lung cancer, the most lethal of all cancers worldwide, arises principally in cells that line the airways. This is generally divided into non-small cell lung cancer (NSCLC), which represents around 85% of cases, and small cell lung cancer, which is more aggressive (98). This disease is associated with risk factors like smoking, carcinogen exposure, and genetic predisposition. The early stage of lung cancer is often asymptomatic; thus, most patients present with advanced disease at diagnosis, and they usually have a poor prognosis (99, 100). Lung cancer is the leading cause of cancer deaths, with more than 2 million new cases reported every year worldwide, especially in developing countries where smoking rates are high. It occurs much more often among men, although it is becoming an increasingly common problem with women (101). Currently, despite increasingly effective surgical and chemotherapeutic treatments, the five-year survival rate is only around 20%, mainly due to the late detection of disease with its aggressive nature (102, 103). There is a growing realization that miRNAs are key players in gene expression closely associated with lung tumor development, progression to advanced disease, and therapeutic resistance (104, 105). All these miRNAs can work as oncomiRs or tumor suppressor genes, influencing key regulators implicated in cell proliferation, apoptosis, and metastasis (106, 107). Considering the ability of miRNAs to serve as both blood-based markers for early detection and prognosis, in addition to being therapeutic targets themselves, they represent a novel target group in personalized treatment strategies for lung cancer (108). For instance, let-7a-5p downregulates the oncogene BCL2L1 by· thereby targeting it for gene silencing through association with a specific mRNA encoding protein activity that affects multiple signaling transcripts and inhibiting cell proliferation and migration/invasive phenotype in lung cancer (109). The miRNA activates the PI3Kγ signaling pathway and induces autophagy and apoptosis in human lung cancer cells (110). Duan et al. utilized let-7a-5p as exosome cargo because high levels of this miRNA in macrophages hinder lung cancer, and the cells also treated A549 NSCLC with BCL2L1 are targets. Surprisingly, let-7a-5p upregulation induced autophagy and cell death in lung cancer cells without triggering apoptosis or pyroptosis (**Figure 2**) (111).

The biological role of TNF-α in lung cancer is paradoxical, being capable of both averting tumor proliferation and also facilitating it (112, 113). On the one hand, this cytokine can mediate inflammation and promote tumor growth through activating transduction pathways that enhance cell survival processes like nuclear factor-κB (NF-κB). It acts alone or together with other agents under various conditions, selectively kills cancer cells by apoptosis cascade clearance mediated through ligating its receptor pathway (114, 115). Jiao et al. identified that TNF-α-stimulated exosomes of PMNs were implicated in sepsis-related ALI, promoting M1 macrophage activation and pyroptosis via the NF-κB signal pathway. Inhibition of this pathway is regulated by exosomal miR-30d-5p directly targeting SOCS1/SIRT1 in macrophages. Knocking down miR-30d-5p decreased the pyroptosis, macrophage activation, and lung injury in sepsis-related acute lung injury (ALI) rats by inhibiting PMN-M interaction via a novel mechanism; these results suggest new potential therapeutic targets for treating sepsis (116).

Moreover, the use of certain microRNAs as diagnostic biomarkers led to progress in the early detection of lung cancer (117). Cazzoli et al. discovered microRNAs able to distinguish lung adenocarcinomas from benign lung conditions with sensitivities and specificities exceeding 90 percent. Their screening test was 97.5% sensitive and 72.0% specific, whereas, for lung adenocarcinoma versus granulomas, their diagnostic assay had a sensitivity of 96.0%, with a specificity of only 60%. This implies a promise of using circulating exosomal miRNAs as early diagnostic markers for lung cancer (118). In addition, Munagala et al. showed that miR-21 and miR-155 exhibited higher levels of the two sequences in serum exosomes from recurrent lung tumors compared to primary ones. These miRNAs are proposed to be potential biomarkers for non-invasive diagnosis of recurrent lung cancer, as their expression patterns in exosomes overlap with those of pathological tissues from primary and metastatic lung tumors. Consequently, miRNAs serve as potential markers of disease progression and relapse monitoring (119). Exosomal miRNAs including let-7a-5p, miR-21, and miR-155 that are released by macrophages influence both the proliferation of tumors and development of drug-resistance as well as function as non-invasive diagnostic and predictive biomarkers that can be detected in serum exosomes (120, 121), which has a practical value in screening and monitoring drug-resistance lung cancer in early diagnosis and the chosen therapeutic response and treatment.

### 5.2 Breast cancer

Breast cancer is the most frequently diagnosed of all cancers among women, with the highest incidence rates in North America and Europe; it originates in ducts (ductal carcinoma) or lobules (lobular carcinoma) (122). With the capacity to metastasize, this cancer can spread all over, and hence its early detection matters (112, 113, 123). The prognosis has substantially improved with the advent of screening methods such as mammography, which allows early detection. There are many treatments for breast cancer, which vary depending on the stage of the disease and its characteristics (124–126). Although the disease is much more common in women over 50, even men can have breast cancer. Age, genetic mutations (e.g., BRCA1/2), family history, reproductive history, and lifestyle factors. Although advancements in detection and treatment have improved survival rates, disparities persist, influenced by geographic, socioeconomic, and racial factors (127).

MiRNAs are involved in the post-transcriptional regulation of gene expression and play a fundamental role in breast cancer by controlling genes related to growth, metastasis, or resistance to therapy. These miRNAs exert oncogenic or tumor suppressor roles modulating key signaling pathways that lead to cancer development (128–130). Due to their potential as biomarkers for diagnosis and prognosis or even therapeutic targets, miRNAs are of great interest in breast cancer research workflow development. In breast cancer, bone marrow stromal macrophages exert a dual effect on both cancer cell dormancy and chemoresistance (131). The M2 macrophages are mainly associated with the induction of dormancy and chemoresistance to cancer cells. The M1 macrophages, however, counteract the dormancy, reactivating the cancer cells and making them more susceptible to chemotherapy (132). This balance is essential in the determination of both dormancy and susceptibility to the treatment of breast cancer in the bone marrow (133). Walker et al. revealed that bone marrow stromal macrophages induced breast cancer cell dormancy, with M2 cells enhancing quiescence and resistance to carboplatin via a gap junctional communication. However, the process was reversed by the M1 cells, which activated NF-κB, increased cellular sensitivity to carboplatin, and improved the survival of the hosts. Driving the modulation of the macrophage phenotype could be the preferred treatment option to control breast cancer dormancy. This will hinder cancer cells from spreading to other parts of the body (134).

Exosomes, as one of the types of microvesicles, are of paramount importance in breast cancer, as they help cells communicate with each other (135). Cells are capable of transferring oncogenic molecules, including several forms of miRNA, proteins, and lipids responsible for the cells’ promotion of tumor growth, invasion, and metastasis (136). Exosomes are of essential importance due to their role in forming the TME (137). According to the study by Yang et al., IL-4-activated macrophages enhance the invasion of breast cancer cells by transferring miR-223. The latter, in turn, raises the invasiveness of cancer cells through the Mef2c-β-catenin pathway. Inhibition of miR-223 in macrophages decreases the suppressive effect of these cells on the invasiveness of breast cancer cells in co-culture, demonstrating the pivotal role of the exosomal transfer of miRNAs in the interaction of macrophages with breast cancer cells that promotes metastasis (138).

One of the leading accelerators of breast cancer development is TAMs. They provide favorable conditions for tumors by enhancing growth, invasion, and metastasis and promoting angiogenesis, immune escape, and tissue remodeling in the TME (139, 140). In most cases, TAMs are oriented toward the M2 type, as it is closely related to the poor prognosis of breast cancer patients (141). The study of Li et al. shows that miR-146a and miR-222 are downregulated, which contributes to tumor progression. Specifically, miR-146a promotes M2 macrophage polarization, which accelerates tumor progression. Secondly, miR-222 inhibits TAM chemotaxis by targeting C-X-C chemokine receptor type 4 (CXCR4) and C-X-C motif chemokine ligand 12 (CXCL12), which ultimately suppresses tumor growth *in vivo*. Thus, miRNAs are a significant factor in regulating TAM functioning and the rate of breast cancer progression (142). MDEs’ miRNAs such as miR-223 and miR-146a affect metastasis and macrophage polarization and hence connect macrophage polarization with tumor aggressiveness and immune evasion (120, 143). Their constancy is contributed by their exosomal state that allows food to nurture them as floating biomarkers and possible agents to reverse this chemoresistance within breast cancer.

### 5.3 Ovarian cancer

Ovarian cancer is a malignancy that starts in the ovaries and often goes undetected until it has spread within the pelvis and abdomen. Therefore, ovarian cancer is the most malignant of gynecologic cancers due to late diagnosis (144, 145). The disease reportedly has several subtypes, including EOC, which is the most common. Generally, however, ovarian cancer is usually manifested by non-specific symptoms such as bloating, pelvic pain, and changes in bowel habits, which makes its detection at early stages quite challenging (146). Moreover, the disease is the eighth most common type of cancer among women worldwide, in addition to being the greatest cause of death from all gynecologic malignancies (147, 148). Ovarian cancer is more commonly reported in Europe and North America than in other parts of the world (149). The disease is mainly encountered among postmenopausal women, with a median age at diagnosis standing at 63 years. Despite the rapid increase in the number of ovarian cancer treatment options, the overall 5-year survival rate remains less than 50% due to its tendency to be diagnosed in late stages (150). In particular, risk factors are common and include age, family history of ovarian cancer, family history of breast cancer, a personal history of breast or ovarian cancer, BRCA1 or BRCA2 mutation, never having given birth, and estrogen hormone therapy (151).

Ovarian cancer depends on oncogenic or tumor suppressor miRNAs that control gene expression involved in tumor development, invasion, and resistance to chemotherapy. Some miRNAs have been strongly linked to poor clinical outcomes in ovarian cancer and have been proposed as early diagnostic or therapeutic targets (152, 153). Li et al. found that exosomal miR-221-3p originating in TAMs drives late EOC progression via CDKN1B inhibition and is selectively enriched in M2 exosomes. miR-221-3p is highly enriched in both the cytoplasm and nucleus of M2 exosomes and appears to stimulate the G1/S phase transmission of EOC cells. The results of this study identify exosomal miR-221-3p as a potential diagnostic serum EOC biomarker and a novel M2-derived target for EOC therapy (57).

**Figure 3:**
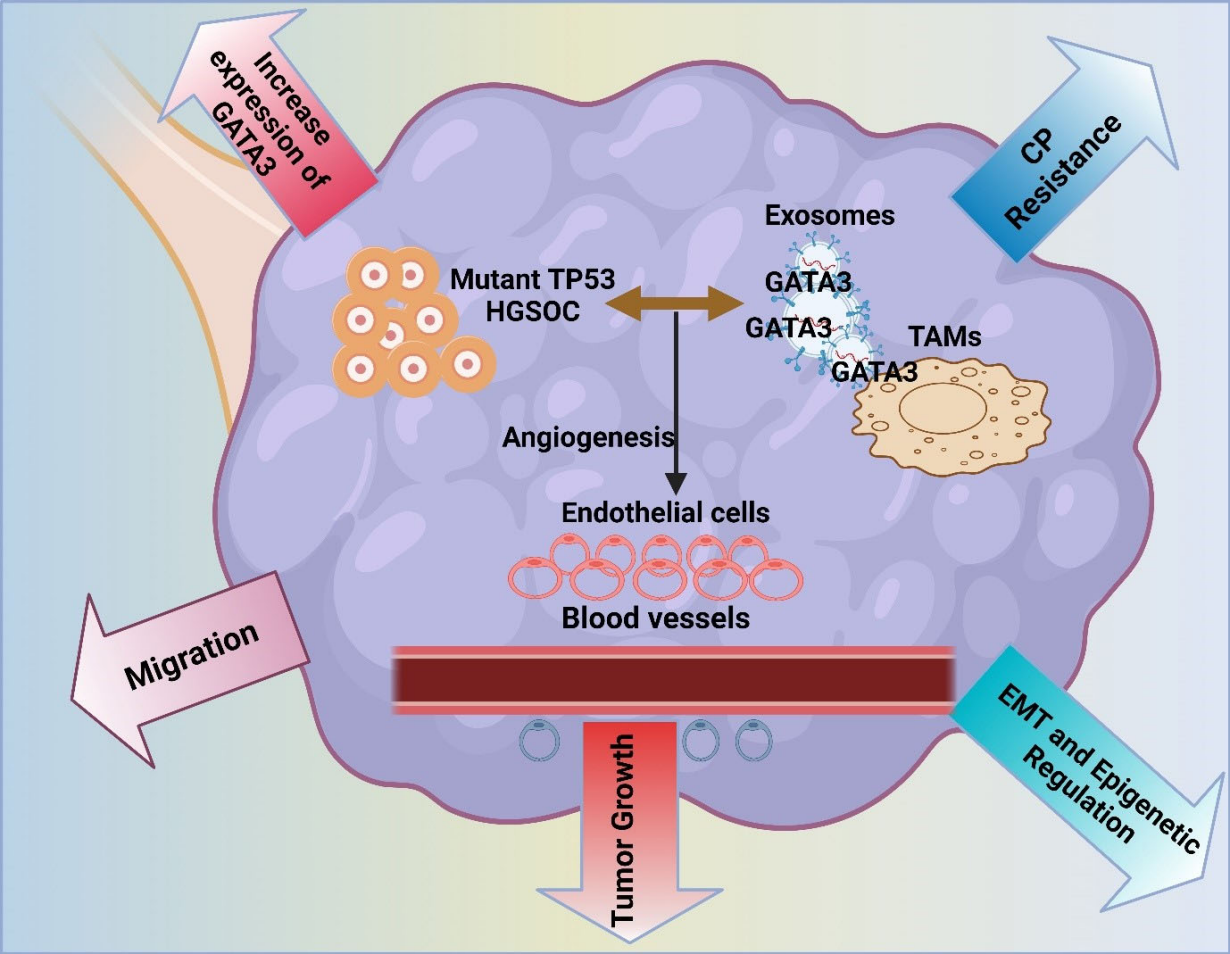
The image illustrates the role of mutant TP53 in high-grade serous ovarian cancer (HGSOC), highlighting GATA3 expression’s impact on tumor growth, angiogenesis, and migration. It also depicts exosome-mediated communication, contributing to chemoresistance, EMT, and epigenetic regulation in the tumor microenvironment.

GATA3 is a transcription factor involved in breast cancer. However, its role in ovarian cancer cells is also emerging as an important finding. These vital factors are often found in cells, which contribute to the progression of the tumor (154). Tumor cells become aggressive by modulating the TME. GATA3 can also activate other associated pathways (155) in a study conducted by El-Arabey et al. GATA3 is demonstrated to be released from TAMs in an exosomal form. This also activates macrophage polarization and its interaction with High-grade serous carcinoma (HGSOC) cells. It facilitates tumor growth and epithelial-mesenchymal transformation. Major tumor-stimulating effects are reduced by the use of siRNA in the GATA3-targeted TAMs. Therefore, GATA3 acts as an important marker for prognosis and a better therapeutic technique for HGSOC (**Figure 3**) (156). The interaction between regulatory T cells and Th17 cells within the TME serves to define the type and the intensity of immune response-resistant EOC, which, as a rule, defines the clinical course and treatment results (157, 158). Tregs play a rather valuable role in boosting tumors, offering a suppressive effect on anti-tumor immunity. In contrast, Th17 cells accumulate in tumors in response to the immune response to regulation (159). The study by Zhou et al. investigated the functions of the exosomal miRNAs from TAMs, which are termed miR-29a-3p and miR-21-5p, since they suppress the CD4+ T cells in a STAT3 manner. This suppresses the balance of the Treg/Th17 and promotes the progression of EOC in an immunosuppressive atmosphere. This remains clear as targeting the exosomes by the miRNAs may be a novel manner of treating EOC (58).

In ovarian cancer, TWEAK double communicates as it promotes tumor progression and immune perturbation. Namely, TWEAK serves to enhance the proliferation, migration, and resistance of cancer cells by activating the NF-κB signaling pathway (160). Moreover, according to Hu et al., exosomes collected by TWEAK-stimulated macrophages impede the metastasis of ovarian cancer by transmitting miR-7 to EOC cells to reduce passage through the EGFR/AKT/ERK1/2 pathway. Using antagomiR-7 transfection, the levels of miR-7 in the macrophages, exosomes, and EOC cells were reduced, and metastasis was enhanced. Studies in a mixed xenograft rodent model showed that TAMs’ exosomal miR-7 prohibits EOC metastasis (66). Besides, Zhu et al. showed that exosomal miR-223 secreted by hypoxic macrophages accelerated the resistance of EOC to chemotherapy by inhibiting the PTEN-PI3K/AKT signaling pathway. Hypoxic exosomes highly expressed miR-223, and they were transferred to EOC to increase its drug resistance. In addition, higher levels of miR-223 contained in the exosomes of patients with EOC were identified in the course of the disease recurrence, which confirms the interaction between macrophages and EOC, which increases the resistance of EOC to chemotherapy (92). M2-macrophage-derived exosomal miR-221-3p, miR-29a-3p, and miR-21-5p are actively amplified to facilitate EOC growth and immune suppression (161). They serve as both clinical agents (correlation in tumor progression prevention) and clinical biomarkers (early detection or monitoring of disease).

### 5.4 Hepatocellular carcinoma

Hepatocellular carcinoma (HCC) is the prime variation of primary liver cancer, usually presenting in the background of chronic liver diseases, such as hepatitis B and C, alcoholic liver disease, and non-alcoholic fatty liver disease (NAFLD). Of note, HCC is characterized by its notorious aggressiveness and extremely poor prognosis (162). Since HCC frequently manifests at an advanced stage, making early detection difficult and curable treatment scarce, this disease is followed by a high rate of death. Regarding the disease frequency, worldwide HCC ranks as the fifth most frequent cancer and the third major cause of cancer death (163). It predominantly affects the male population and has been notably recognized in East Asia and Sub-Saharan African regions, wherein hepatitis B and C infections are common. Nevertheless, in Western countries, this phenomenon is continuously increasing as a result of the burgeoning rates of obesity, diabetes, and NAFLD (164, 165). Notably, the function of miRNAs in HCC is pivotal. It is related to their ability to control gene expression associated with tumor development, progression, and metastasis in either a stimulatory or repressive manner (166).

CD90 is one of the important markers in HCC, and it is Thymus cell antigen 1 (Thy-1), a stem cell antigen that plays an essential role as a marker for cancer stem cells (CSCs). A variety of lines of evidence suggest that CD90+ cells are associated with tumor initiation, progression, and resistance to therapy. CD90+ cells in HCCs exhibit higher self-renewal, invasion, and metastasis properties (167, 168). Wang et al. demonstrated that exosomes from TAMs promote HCC cell proliferation and enhance the relative CSC traits by delivering miR-125a/b. The down-regulation of miR-125a/b in TAM-derived exosomes enhanced CSC traits associated primarily targeting the stem cell marker CD90. These results suggest that miR-125a and miR-125 b play a critical role in the regulation of CSCs in HCC through TAM-derived exosomes (169). Apart from miR-125a/b, miR-142 and miR-223 transfer between the cells substantially contributes to HCC (170). Aucher et al. showed that human macrophages transfer miR-142 and miR-223 to HCC cells via gap junctions. The transfer inhibits the proliferation of HCC since it regulates the expression of the key proteins, including stathmin-1 and insulin-like growth factor 1 (IGF-1) receptor (IGF1R); thus, a novel defense mechanism of immune cells from tumor proliferation. Multiple mechanisms and processes keep cell proliferation under control, and some of them have been discovered only recently, such as the transfer of small RNA molecules between cells (171).

Alcoholic liver disease is a disorder that is caused by excessive consumption of alcohol. This results in damage to the liver, which could range from fatifying of liver to more severe forms of the disorder, such as alcoholic hepatitis or cirrhosis (172). The chronic consumption of alcohol disrupts the normal functions of the liver cells and is associated with inflammation, which may cause an individual to develop scar tissue on the liver. It increases the risk of liver failure and death and is regarded as a major cause of liver-related incidences of morbidity and mortality in most parts of the world (172). Babuta et al. found that alcohol disrupts autophagy in alcoholic liver disease (ALD) by impairing the functions of autophagosomes and lysosomes. The latter is attributed to the downregulation of Lysosome-Associated Membrane Protein 1 & Protein 2 (LAMP1 and LAMP2). This is a result of miR-155, which targets key oncogenes in the process and causes an increase in the production of exosomes. Notably, the mice that lacked the miR-155 molecule were not affected by the effects, and this proves its role in the disruption of autophagy and the release of exosomes by alcohol in ALD (173). TAM-derived exosomal miR-125a/b, miR-142, and miR-223 control the expression of cancer-stem-cell-associated genes like CD90 that boost tumor recurrence and resistance in HCC (174). Their practical usefulness and their identification in plasma justify their possible use as biomarkers that predict relapse and treat therapeutic nodes in precision-based management of HCC.

### 5.5 Pancreatic cancer

Pancreatic cancer is a highly aggressive malignancy that originates in the pancreatic tissues and is often detected late, as it is asymptomatic and has rapid progress and a lack of response to any treatments (175). This is the unfinished business of most malignant neoplasms in the pancreas and perhaps one of the most lethal types of cancer. Pancreatic ductal adenocarcinoma (PDAC) is the most common form of pancreatic cancer (176). Risk factors associated with this blockage include cigarette smoking, chronic pancreatitis, being overweight, and having a family history of pancreatic cancer. There are only a several drugs to deal with this diagnosis, which is why the five-year percentile has a very low rate of 5-10% pancreatic cancer (177). Compared to other types of cancer, this one is the twelfth different cancer.

On the other hand, it has a high mortality rate and occupies the seventh place among the deadliest forms of neoplasms. The biggest number of incidence falls in prosperous countries where a large number of men were affected by it (178). People who are obese and have diabetes are also in the high-risk group. One of the critical roles of miRNAs in pancreatic cancer is the involvement in signaling pathways that control the growth, metastasis, and drug resistance of tumors. This is achieved by miRNAs either as oncogenes or as tumor-suppressive genes (179). Binenbaum et al. discovered that miR-365, which is found in TAMs-derived exosomes in PDAC, impairs gemcitabine activity, which is a drug used in chemotherapy, thus inducing cancer cell resistance to this medicine. The study showed that restoring the latter’s sensitivity was possible by blocking miR-365, which provides yet more evidence for the fact that MDEs are among the primary regulators of chemotherapy resistance in PDAC (94).

X-linked inhibitor of apoptosis protein (XIAP) is also a critical factor in pancreatic cancer. It inhibits apoptosis and promotes cell survival. Hence, the XIAP’s overexpression in cancer cells guarantees survival despite chemotherapy and other treatments (180). Overexpression of the factor is associated with poor prognosis and the aggressiveness of cancer. Interestingly, XIAP seems to be a practical therapeutic target in pancreatic cancer (181) in the study. Yin et al. show that M2 macrophage-derived exosomes promote pancreatic cancer by transmitting long non-coding RNA SBF2-AS1 that suppresses MiR-122-5p and upregulates XIAP. Suppression of XIAP restrains the tumor from growing, helping treat the disease (182). Similarly, Yin et al., revealed that exosomes in M2 TAMs consist of miR-501-3p to advance PDAC via targeting the suppression gene TGFBR3. This downregulation, in turn, activates the TGF-β signaling pathway that will foster tumor growth and metastasis. Depletion of miR-501-3p in these exosomes acted to suppress tumorigenesis and metastasis, together with their upstream TGF-β signaling pathway, might be viable therapeutic targets for PDAC treatment (56). Macrophage-derived exosomal miR-365 silenced the effect of gemcitabine by interfering with nucleotide metabolism in PDAC, and miR-501-3p and lncRNA SBF2-AS1 changed TGF-β and XIAP to promote chemoresistance (94). These results reveal MDE cargoes as potent drug resistance mediators and promising therapeutic targets tested in *in vivo* xenograft studies.

### 5.6 Colorectal cancer

CRC is a type of cancer that develops in the colon or rectum. It is often preceded by the growth of benign polyps, which may later become malignant. It is the third most common cancer and the second leading cause of cancer mortality globally (183). The common symptoms experienced are changes in defecation rhythm, bloody stool, and abdominal pain. CRC is monitored by colonoscopy, and when detected, early survival rates are high (184). It is treated with surgery, chemotherapy, and radiation, often in combination. The incidence rates of CRC are third in the most often diagnosed cancers and second in terms of deaths worldwide, with higher rates in the more developed areas, particularly North America, Europe, and Australia (185). There are numerous risk factors, such as age, family history, diet, smoking and alcohol consumption, obesity, and inflammatory bowel diseases. There has been a downward trend in the mortality rates associated with CRC directly linked to the implementation of screening programs, as the introduction of early detection methods has been decisive (186).

MiRNAs play an essential role in CRC by controlling the expression of genes. These genes are responsible for the growth, metastasis, and response to treatment of the tumor. When miRNAs are not functioning properly, they can become oncogenic or mutated (187). Undeveloped miRNAs have attracted consideration as potential indicators for the first detection of CRC. They are additionally appealing targets for therapeutic intervention and TSR catalysts. There has been an observation that miR-15a and miR-16 are significantly under-expressed in CRC tissues in contrast to the normal mucosa (188). According to the research study of Xiao et al., the low expression of these miRNAs is dependent on the advanced disease degree, poor histological evaluation, and the presence of nodes. The cumulative low level of these two miRNAs can securely guarantee a deficient general and disease-free existence of CRC patients. On this note, the delivery of these miRNAs, which are not well controlled, to the agitated tumors through exosomes may be difficult. The miR-15a and miR-16 are often used as markers for treatment and recovery (189).

The p53 protein is a key player in the mechanism of colon cancer as a tumor suppressor, orchestrating the arrest of the cell cycle, DNA repair, or apoptosis, when necessary, in response to cellular stress or damage to DNA. However, the TP53 gene, which encodes the p53 protein, is mutated in numerous cases of colon cancer, thus losing its function of suppressing tumors (190). In their studies, Cooks et al. found that colon cancer cells with gain-of-function mutp5–3 secrete exosomes containing a high concentration of miR-1246. These exosomes reprogram the TAMs into an anti-inflammatory and cancer-promoting state in CRC through the action of miR-1246. Thus, miR-1246 sustains the immunosuppression, as well as the activity of TGF-β, thus promoting the inflammatory state, as well as the progression of cancer, and poor survival of patients with CRC (191). One of the proteins is ZC3H12B, which is a tumor suppressor in colon cancer, regulating inflammation and cell proliferation (192). The downregulation or loss of ZC3H12B in colon cancer cells results in a more aggressive tumor, increased growth, invasion, and worse prognosis. Such a suppression degrades pro-inflammatory mRNAs, reducing the inflammatory environment that allows cancer to progress (193). Another protein that was identified by Ma et al. is M2 macrophage-derived exosomal miR-155-5p. It fosters immune escape in CRC, interacting with ZC3H12B, degrading its expression, and raising IL-6 levels. As a result, CRC proliferation and anti-apoptotic were supported, and immune escape was achieved. It is possible to regard that such a miR-155-5p in exosomes might be one of the CRC progressors and an anti-cancer target (96). MDE-mediated miR-155-5p and miR-1246 drive immune escape and pro-inflammatory signaling in CRC by regulating ZC3H12B and TGF-β cascade (194). Their consistent detection in patient exosomes underscores their value as liquid biopsy biomarkers and targets to re-sensitize tumors to therapy.

### 5.7 Other cancers

MDEs have shown potential to be a great inducer in numerous cancers other than lung, pancreatic, colorectal, ovarian, and breast cancers. These exosomes promote tumorigenesis by promoting disease progression. Moreover, MDEs transfer oncogenic miRNAs and proteins that have implications for the upregulation of TME alteration-related genes (195). The upregulated genes promote glioblastoma (GBM) due to the interaction and alteration of the molecular pathways conducive to GBM growth and enhancement of therapy resistance. These exosomes were shown to promote gastric cancer (GC) by activating new metastasis-related genes (196). It seems that MDEs promote cancer progression and metastasis in all organs of a living cell, although they have not been isolated from all organs to date. In EC, miRNAs seem to play a significant role in shifting gene equilibrium to produce changes in the rate of cancer development (197).

Moreover, the altered miRNAs may act as oncogenes or tumor suppressors. Changes in the pathway characteristic of these types of miRNAs, such as cell proliferation, apoptosis, and invasion, have made the miRNAs popular in the hunt for diagnostic biomarkers and treatments (198). Mi et al. showed that M2 macrophage-derived exosomes are involved in the migration and invasion of EC. The exosomes contained the long non-coding RNA AFAP1-AS1 that inhibited miR-26a and promoted ATF2. EC was found to be able to migrate and invade due to the expression of ATF2 alone or together with the miRNA. The results showed that a therapeutic strategy could be initiated by targeting this signaling pathway, supporting more advanced EC (62). MDEs are involved in GC via the transfer of miRNAs that regulate tumor growth, invasion, and metastasis.

As the miRNAs act on the gene expression of the TME, they are one target for cancer therapeutic approaches (199). Zheng et al. discovered that TAMs activate the migration of GC through exosomes by polarizing into M2 macrophages. This exosome transfer not only involves lipid transfer but also the transfer of Apolipoprotein E (ApoE) and specific miRNAs. After the delivery of the M2-derived exosomes into the cancer cells, their migration ability is enhanced by the PI3K-AKT signaling pathway. The cancer cells from Apoe-/- mice lacked the effect of the exosomes on their migration ability, and this indicates the importance of ApoE and miRNAs in driving the exosomal transfer by TAMs in the progression of cancer (200). Li et al. found that miR-16-5p loaded into exosomes derived from M1 macrophages suppresses the development of GC via the activation of T-cell-dependent immunity by targeting programmed death-ligand 1 expression in GC cells. The delivery of miR-16-5p from M1 macrophages to GC cells induced an immune response against the tumor *in vitro* and *in vivo*, specifically. Hence, the authors concluded the proposal that M1 macrophages could serve as a cellular treatment agent for GC by facilitating miR-16-5p delivery in exosomes (201). Gao et al. discovered that macrophage-derived exosomal miR-223 inhibited tumor-suppressive ubiquitin ligase substrate specificity of F-box and WD-40 repeat domain-containing 7 to promote doxorubicin resistance in GC. The transfer of miR-223 from macrophages to GC cells was found to occur using exosomes, and knockdown of miR-223 in macrophages appeared to reduce the resistance. As for clinical settings, the presence of high levels of miR-223 in both GC tissues and plasma exosomes has been linked to doxorubicin resistance. Therefore, due to this link, targeting exosome-mediated miR-223 transfer is likely to become a helpful therapy for GC patients (202).

**Figure 4:**
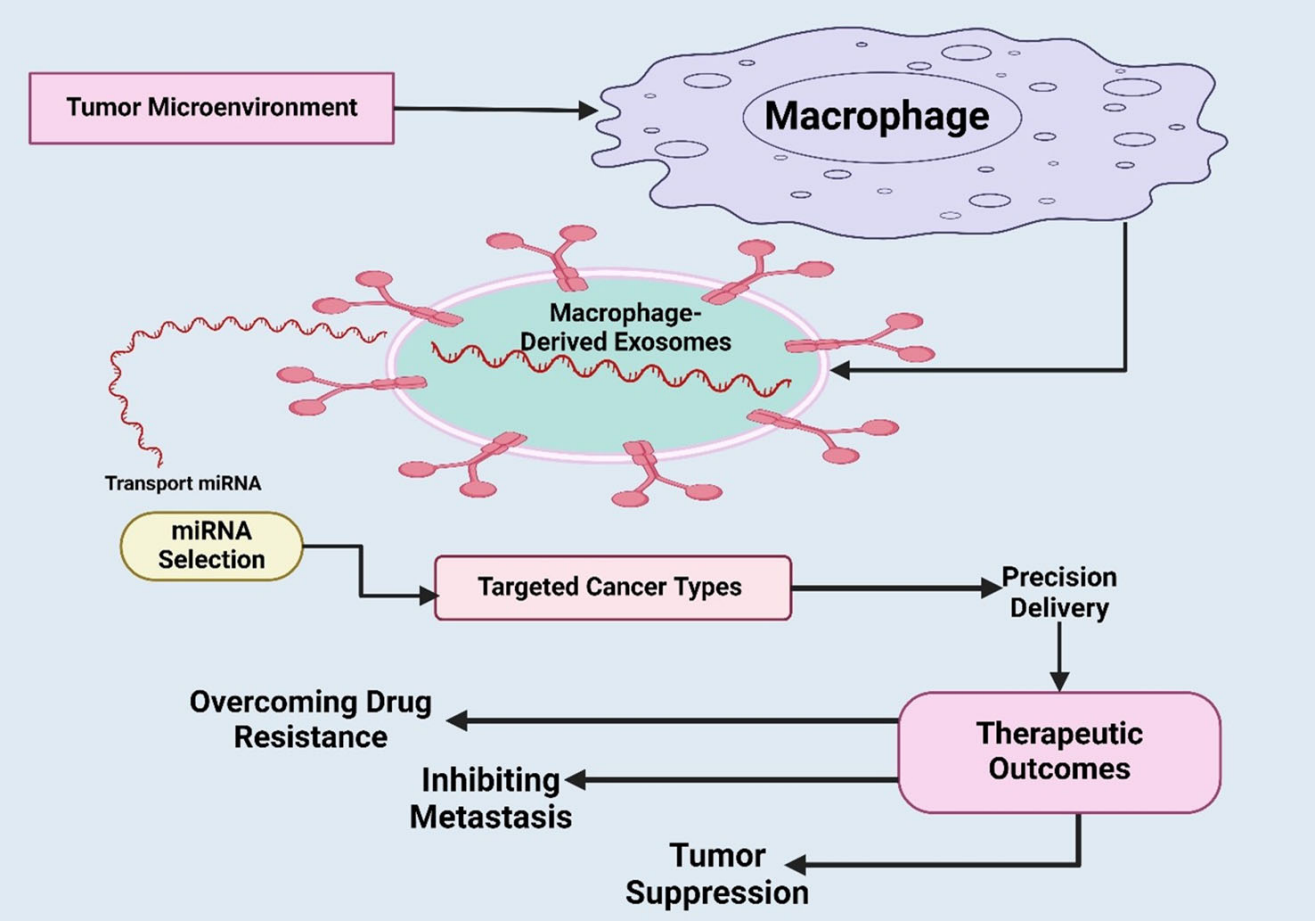
Illustration of dynamic interplay between macrophages and the tumor microenvironment via macrophage-derived exosomes were depicted. It also highlights how selected miRNAs are transported within exosomes to specific cancer targets, enabling precision delivery of therapeutic molecules. Overall, it underscores the translational significance of exosome-based strategies for targeted cancer therapy.

In GBM, miRNAs serve vital functions that facilitate the regulation of targeted genes, such as gene growth, invasion, and resistance to therapy. MiRNAs could be oversensitive or work as inhibitors of these processes, leading to their function as oncogenes or tumor suppressors that can further control major pathways and be involved in GBM (203). The role of miRNAs as biomarkers for early diagnosis, prognosis, and therapeutic targets is promising for those affected by GBM (204). Moreover, it was emphasized by Qian et al. that hypoxic glioma-derived exosomes significantly promote the M2 polarization of macrophages, resulting in enhanced glioma growth, movements, and aggressive invasion. The microRNA sequencing identified miR-1246 as the leading miRNA in H-GDEs, positively correlated with the activity of the STAT3 signaling pathway operated by the targeting recognition of TERF2IP. It was concluded that miR-1246 levels in the cerebrospinal fluid of GB patients are novel targeted chemotherapy antitumor factors (205). In other malignancies, in GC, GBM, and EC, the MDEs miRNAs, e.g., miR-223, miR-16-5p, and miR-1246, coordinate metastasis, chemoresistance, and immune modulation. The fact that they are retained regulators in a wide variety of tumor types points to their potential in becoming pan-cancer exosomal biomarkers and pan-cancer targets of therapeutic interest (206). The dynamic interplay between macrophages and the tumor microenvironment via macrophage-derived exosomes was depicted (**Figure 4**).

## 6 Limitations, safety concerns, and translational barriers of engineered MDEs

Although MDEs containing therapeutic miRNAs hold enormous potential, a series of biological and translational limitations need to be overcome before using these in clinical practice.

First, the problem of off-target effects must stay significant: the non-target tissues have the potential to absorb exosomal miRNAs, causing unwanted gene silencing and possibly a change in physiological or immune functions (207). On the same note, MDEs have inherent immunomodulatory functions, potentially leading to undesirable immunosuppression or a balance in cytokines, thus facilitating tumor immune evasion over regression (208).

In terms of bioengineering, heterogeneity of exosome isolation and miRNA loading capacity does not help with the reproducibility and dose standardization in production batches. Moreover, the large-scale production of exosomes, the absence of potent purification procedures, and the inconsistency of switching between the polarization states of the macrophages considerably affect therapy consistency (209). A further significant roadblock in the translation is related to biodistribution and clearance: when injected intravenously, MDEs are quickly taken away by the liver and the spleen, minimizing their accumulation and therapeutic efficacy to tumor properties (210, 211).

Besides, the regulatory environment of exosome-based therapeutics remains to be developed, and there is a lack of agreed quality control standards, long-term safety evaluation, and immunogenicity examination. Therefore, even with the promising preclinical research, the development of engineered MDE-miRNA systems into the clinic would demand stringent pharmacokinetic, toxicological, and immunological studies in a variety of animal models and controlled early-phase human trials. The standardized manufacturing protocols, specific surface engineering, and the selection of the miRNA will play a key role in the implementation of reproducible, safe, and effective MDE-based cancer therapies. Also, we have summarized the overview of macrophage-derived exosome engineering and modification for cancer therapeutics and diagnostics in **Table 2**.

**Table 2:** Overview of macrophage-derived exosome engineering and modification for cancer applications.

Resources	M1/M2/M0	Surface engineering	Loading molecular	Loading method	Application	Study model	Reference
Lung Cancer	M1/M2	Exosome engineering	miR-30d-5p, let-7a-5p	Exosome loading	Treatment of NSCLC, Sepsis-related ALI, Autophagy induction, Apoptosis regulation	*in vitro* (A549 NSCLC cells)	(111)
Lung Cancer	M1	Exosome modification	miR-21, miR-155	Exosome loading	Diagnostic biomarker for recurrent lung cancer		(119)
Lung Cancer	M1	Exosome engineering	miR-30d-5p, SOCS1/SIRT1	Exosome loading	M1 macrophage activation, Pyroptosis regulation		(116)
Breast Cancer	M2	Exosome engineering	miR-223	Exosome loading	Promoting breast cancer cell invasion and metastasis	*in vitro* (macrophage–BC co-culture)	(138)
Breast Cancer	M2	Exosome modification	miR-146a, miR-222	Exosome loading	Tumor progression, Immune escape		(142)
Ovarian Cancer	M2	Exosome engineering	miR-221-3p	Exosome loading	Progression of EOC, Diagnostic serum biomarker	*in vitro*; clinical (serum exosomes)	(57)
Ovarian Cancer	M2	Exosome modification	miR-29a-3p, miR-21-5p	Exosome loading	Suppression of T cells, EOC progression	*in vitro*; clinical (patient plasma exosomes)	(58)
Hepatocellular Carcinoma (HCC)	M2	Exosome engineering	miR-125a/b	Exosome loading	Regulation of cancer stem cells, Inhibition of proliferation	*in vitro* (HCC cell lines)	(169)
Hepatocellular Carcinoma	M2	Exosome modification	miR-142, miR-223	Exosome loading	Inhibition of proliferation, Immune defense		(171)
Pancreatic Cancer	M2	Exosome modification	miR-365	Exosome loading	Chemotherapy resistance, Tumor progression	*in vitro* only (no *in vivo* xenograft)	(94)
Pancreatic Cancer	M2	Exosome modification	miR-501-3p	Exosome loading	Tumor progression, Activation of TGF-β signaling pathway	*in vitro* only (no *in vivo* xenograft)	(182)
Colorectal Cancer	M2	Exosome engineering	miR-1246	Exosome loading	Immune escape, Inflammation, Tumor progression		(191)
Colorectal Cancer	M2	Exosome engineering	miR-155-5p	Exosome loading	Immune escape, Tumor progression	*in vitro* (CRC cell lines)	(96)
Glioblastoma	M2	Exosome modification	miR-1246	Exosome loading	Tumor growth, Invasion, Therapy resistance		(205)
Gastric Cancer	M2	Exosome engineering	miR-223	Exosome loading	Drug resistance, Tumor progression	*in vitro*; clinical (plasma/tissue association with doxorubicin resistance)	(202)
Gastric Cancer	M2	Exosome modification	miR-223	Exosome loading	Tumor progression, Metastasis	*in vitro*; *in vivo* (Apoe−/− mice)	(200)

## 7 Conclusion and future prospects

Collectively, these findings support clear near-term opportunities for translational studies while highlighting specific avenues for optimization and clinical validation. MDEs have a vital function in cancer biology by serving as intermediaries for communication between macrophages and tumor cells. These exosomes play a crucial role in several processes that are vital for the advancement of cancer, such as stimulating the development of tumors, aiding in the spread of cancer to other parts of the body, causing resistance to drugs, and influencing the immune response. MDEs can greatly modify the behavior of cancer cells and the TME by transporting specific cargo, especially miRNAs. The results presented in this context demonstrate that MDEs include miRNAs that may either facilitate or impede cancer development, depending on their unique composition. For instance, the introduction of cancer-causing miRNAs via MDEs may amplify the development, invasion, and resistance to the treatment of tumors. In contrast, miRNAs that decrease tumor growth can hinder these processes. The ability to manipulate MDEs for the specific administration of therapeutic miRNAs presents new opportunities for precision medicine in the field of cancer, providing enhanced and individualized therapy alternatives.

Advances in the MDE-miRNA regimens rely on standardized work on the isolation and characterization of exosomes. Spike-in controls, cross-platform reproducibility, appropriate normalization, and common QC standards for miRNA profiling should be implemented. To be delivered safely, engineered MDEs need constructive evaluation of biodistribution, immunogenicity, complement activation, thrombogenicity, and off-target effects, preferably on two vertebrates before clinical translation. Embracing these standards will make it possible to achieve a realistic, reproducible, and safe route to clinical testing of MDE-miRNA-based therapies.

Nevertheless, several critical constraints still need to be addressed to facilitate clinical translation. These include improving the precision of miRNA loading into MDEs and enhancing their tumor-specific delivery while minimizing off-target effects. Future studies should focus on optimizing ESCRT-dependent and -independent sorting pathways to enable selective and reproducible miRNA encapsulation. Parallel efforts should evaluate engineered MDEs functionalized with tumor-homing ligands or stimuli-responsive coatings in preclinical and early-phase (Phase I) clinical trials to assess biodistribution, immunogenicity, and therapeutic efficacy. Moreover, integrating multi-omics analyses with bioengineering and nanotechnology platforms could allow real-time monitoring of exosomal cargo loading and release kinetics, offering a rational framework for precision MDE-based therapeutics. Addressing these challenges through interdisciplinary strategies will accelerate the safe and effective translation of MDE-miRNA delivery systems into personalized cancer care.

## References

[1] Abe C Bhaswant M Miyazawa T Miyazawa T . The potential use of exosomes in anti-cancer effect induced by polarized macrophages. Pharmaceutics. (2023) 15:1024. doi: 10.3390/pharmaceutics15031024, PMID: 36986884 PMC10054161

[2] Zhang Z Liu L Ti H Chen M Chen Y Du D . Synovial fibroblast derived small extracellular vesicles miRNA15–29148 promotes articular chondrocyte apoptosis in rheumatoid arthritis. Bone Res. (2025) 13:61. doi: 10.1038/s41413-025-00430-3, PMID: 40506465 PMC12162823

[3] Lu JY Guo Z Huang WT Bao M He B Li G . Peptide-graphene logic sensing system for dual-mode detection of exosomes, molecular information processing and protection. Talanta. (2024) 267:125261. doi: 10.1016/j.talanta.2023.125261, PMID: 37801930

[4] Baig MS Roy A Rajpoot S Liu D Savai R Banerjee S . Tumor-derived exosomes in the regulation of macrophage polarization. Inflammation Res. (2020) 69:435–51. doi: 10.1007/s00011-020-01318-0, PMID: 32162012

[5] Tai YL Chen KC Hsieh JT Shen TL . Exosomes in cancer development and clinical applications. Cancer Sci. (2018) 109:2364–74. doi: 10.1111/cas.13697, PMID: 29908100 PMC6113508

[6] Huang H Huang F Liang X Fu Y Cheng Z Huang Y . Afatinib reverses EMT via inhibiting CD44-stat3 axis to promote radiosensitivity in nasopharyngeal carcinoma. Pharm (Basel). (2022) 16:37. doi: 10.3390/ph16010037, PMID: 36678534 PMC9864417

[7] Khan Y Hussain MS Ramalingam PS Fatima R Maqbool M Ashique S . Exploring extracellular RNA as drivers of chemotherapy resistance in cancer. Mol Biol Rep. (2025) 52:142. doi: 10.1007/s11033-025-10263-2, PMID: 39836259

[8] Ammendola M Curcio S Ammerata G Luposella M Battaglia C Laface C . Macrophages in tumor microenvironment: from molecular aspects to clinical applications. Eurasian J Med Oncol. (2023) 7:201. doi: 10.14744/ejmo.2023.26480

[9] Zhang Y Zhu L Li X Ge C Pei W Zhang M . M2 macrophage exosome-derived lncRNA AK083884 protects mice from CVB3-induced viral myocarditis through regulating PKM2/HIF-1α axis mediated metabolic reprogramming of macrophages. Redox Biol. (2024) 69:103016. doi: 10.1016/j.redox.2023.103016, PMID: 38160539 PMC10792748

[10] Beeraka NM Doreswamy SH Sadhu SP Srinivasan A Pragada RR Madhunapantula SV . The role of exosomes in stemness and neurodegenerative diseases-chemoresistant-cancer therapeutics and phytochemicals. Int J Mol Sci. (2020) 21:6818. doi: 10.3390/ijms21186818, PMID: 32957534 PMC7555629

[11] Chen Q Li Y Gao W Chen L Xu W Zhu X . Exosome-mediated crosstalk between tumor and tumor-associated macrophages. Front Mol Biosci. (2021) 8:764222. doi: 10.3389/fmolb.2021.764222, PMID: 34722637 PMC8549832

[12] Fabris L Sato K Alpini G Strazzabosco M . The tumor microenvironment in cholangiocarcinoma progression. Hepatology. (2021) 73 Suppl 1:75–85. doi: 10.1002/hep.31410, PMID: 32500550 PMC7714713

[13] Chatterjee B Saha P Bose S Shukla D Chatterjee N Kumar S . MicroRNAs: as critical regulators of tumor- associated macrophages. Int J Mol Sci. (2020) 21:7117. doi: 10.3390/ijms21197117, PMID: 32992449 PMC7582892

[14] Song Y Huang Y Zhou F Ding J Zhou W . Macrophage-targeted nanomedicine for chronic diseases immunotherapy. Chin Chem Lett. (2022) 33:597–612. doi: 10.1016/j.cclet.2021.08.090

[15] Abd-Aziz N Kamaruzman NI Poh CL . Development of microRNAs as potential therapeutics against cancer. J Oncol. (2020) 2020:8029721. doi: 10.1155/2020/8029721, PMID: 32733559 PMC7378626

[16] Fang X Lan H Jin K Qian J . Pancreatic cancer and exosomes: role in progression, diagnosis, monitoring, and treatment. Front Oncol. (2023) 13:1149551. doi: 10.3389/fonc.2023.1149551, PMID: 37287924 PMC10242099

[17] Iqbal MJ Javed Z Sadia H Mehmood S Akbar A Zahid B . Targeted therapy using nanocomposite delivery systems in cancer treatment: highlighting miR34a regulation for clinical applications. Cancer Cell Int. (2023) 23:84. doi: 10.1186/s12935-023-02929-3, PMID: 37149609 PMC10164299

[18] Chen Q Shan X Shi S Jiang C Li T Wei S . Tumor microenvironment-responsive polydopamine-based core/shell nanoplatform for synergetic theranostics. J Mater Chem B. (2020) 8:4056–66. doi: 10.1039/D0TB00248H, PMID: 32270145

[19] Chakrabortty A Patton DJ Smith BF Agarwal P . miRNAs: potential as biomarkers and therapeutic targets for cancer. Genes (Basel). (2023) 14:1375. doi: 10.3390/genes14071375, PMID: 37510280 PMC10378777

[20] Yan J Liu H Yang W Liu N Wang J Li Z . Small-molecule-induced liquid-liquid phase separation suppresses the carcinogenesis of β-catenin. Nat Commun. (2025) 16:5997. doi: 10.1038/s41467-025-61112-6, PMID: 40593772 PMC12217888

[21] Arya SB Collie SP Parent CA . The ins-and-outs of exosome biogenesis, secretion, and internalization. Trends Cell Biol. (2024) 34:90–108. doi: 10.1016/j.tcb.2023.06.006, PMID: 37507251 PMC10811273

[22] Lee YJ Shin KJ Jang HJ Ryu JS Lee CY Yoon JH . GPR143 controls ESCRT-dependent exosome biogenesis and promotes cancer metastasis. Dev Cell. (2023) 58:320–334.e328. doi: 10.1016/j.devcel.2023.01.006, PMID: 36800996

[23] Mashouri L Yousefi H Aref AR Ahadi AM Molaei F Alahari SK . Exosomes: composition, biogenesis, and mechanisms in cancer metastasis and drug resistance. Mol Cancer. (2019) 18:75. doi: 10.1186/s12943-019-0991-5, PMID: 30940145 PMC6444571

[24] Wen B Tao R Liu Y Zhang Z . Investigating the role of exosomal microRNA-5703 in modulating tumor-associated endothelial cells in lung cancer. Cytojournal. (2024) 21:77. doi: 10.25259/Cytojournal_99_2024, PMID: 39917007 PMC11801689

[25] Raposo G Stoorvogel W . Extracellular vesicles: exosomes, microvesicles, and friends. J Cell Biol. (2013) 200:373–83. doi: 10.1083/jcb.201211138, PMID: 23420871 PMC3575529

[26] Wei H Chen Q Lin L Sha C Li T Liu Y . Regulation of exosome production and cargo sorting. Int J Biol Sci. (2021) 17:163–77. doi: 10.7150/ijbs.53671, PMID: 33390841 PMC7757038

[27] Zhang L Yu D . Exosomes in cancer development, metastasis, and immunity. Biochim Biophys Acta Rev Cancer. (2019) 1871:455–68. doi: 10.1016/j.bbcan.2019.04.004, PMID: 31047959 PMC6542596

[28] Zhang X Xu Y Ma L Yu K Niu Y Xu X . Essential roles of exosome and circRNA_101093 on ferroptosis desensitization in lung adenocarcinoma. Cancer Commun (Lond). (2022) 42:287–313. doi: 10.1002/cac2.12275, PMID: 35184419 PMC9017758

[29] Zhao S Mi Y Guan B Zheng B Wei P Gu Y . Tumor-derived exosomal miR-934 induces macrophage M2 polarization to promote liver metastasis of colorectal cancer. J Hematol Oncol. (2020) 13:156. doi: 10.1186/s13045-020-00991-2, PMID: 33213490 PMC7678301

[30] Luo G Zhou Z Cao Z Huang C Li C Li X . M2 macrophage-derived exosomes induce angiogenesis and increase skin flap survival through HIF1AN/HIF-1α/VEGFA control. Arch Biochem Biophys. (2024) 751:109822. doi: 10.1016/j.abb.2023.109822, PMID: 38030054

[31] Villarroya-Beltri C Gutiérrez-Vázquez C Sánchez-Cabo F Pérez-Hernández D Vázquez J Martin-Cofreces N . Sumoylated hnRNPA2B1 controls the sorting of miRNAs into exosomes through binding to specific motifs. Nat Commun. (2013) 4:2980. doi: 10.1038/ncomms3980, PMID: 24356509 PMC3905700

[32] Shurtleff MJ Temoche-Diaz MM Karfilis KV Ri S Schekman R . Y-box protein 1 is required to sort microRNAs into exosomes in cells and in a cell-free reaction. Elife. (2016) 5:e19276. doi: 10.7554/eLife.19276, PMID: 27559612 PMC5047747

[33] Koppers-Lalic D Hackenberg M Bijnsdorp IV Van Eijndhoven M Sadek P Sie D . Nontemplated nucleotide additions distinguish the small RNA composition in cells from exosomes. Cell Rep. (2014) 8:1649–58. doi: 10.1016/j.celrep.2014.08.027, PMID: 25242326

[34] Kosaka N Iguchi H Hagiwara K Yoshioka Y Takeshita F Ochiya T . Neutral sphingomyelinase 2 (nSMase2)-dependent exosomal transfer of angiogenic microRNAs regulate cancer cell metastasis. J Biol Chem. (2013) 288:10849–59. doi: 10.1074/jbc.M112.446831, PMID: 23439645 PMC3624465

[35] Han Q Zhao H Jiang Y Yin C Zhang J . HCC-derived exosomes: critical player and target for cancer immune escape. Cells. (2019) 8:558. doi: 10.3390/cells8060558, PMID: 31181729 PMC6627799

[36] Krylova SV Feng D . The machinery of exosomes: biogenesis, release, and uptake. Int J Mol Sci. (2023) 24:1337. doi: 10.3390/ijms24021337, PMID: 36674857 PMC9865891

[37] Zhou Z Sui X Cao Z Li X Qing L Tang J . Substance P promote macrophage M2 polarization to attenuate secondary lymphedema by regulating NF-kB/NLRP3 signaling pathway. Peptides. (2023) 168:171045. doi: 10.1016/j.peptides.2023.171045, PMID: 37507091

[38] Jeffries J Zhou W Hsu AY Deng Q . miRNA-223 at the crossroads of inflammation and cancer. Cancer Lett. (2019) 451:136–41. doi: 10.1016/j.canlet.2019.02.051, PMID: 30878527 PMC6441621

[39] Lee YJ Shin KJ Chae YC . Regulation of cargo selection in exosome biogenesis and its biomedical applications in cancer. Exp Mol Med. (2024) 56:877–89. doi: 10.1038/s12276-024-01209-y, PMID: 38580812 PMC11059157

[40] Jo H Shim K Jeoung D . Exosomes: diagnostic and therapeutic implications in cancer. Pharmaceutics. (2023) 15:1465. doi: 10.3390/pharmaceutics15051465, PMID: 37242707 PMC10223086

[41] Blanc L Vidal M . New insights into the function of Rab GTPases in the context of exosomal secretion. Small GTPases. (2018) 9:95–106. doi: 10.1080/21541248.2016.1264352, PMID: 28135905 PMC5902209

[42] Khalaf K Hana D Chou JT Singh C Mackiewicz A Kaczmarek M . Aspects of the tumor microenvironment involved in immune resistance and drug resistance. Front Immunol. (2021) 12:656364. doi: 10.3389/fimmu.2021.656364, PMID: 34122412 PMC8190405

[43] Pollet H Conrard L Cloos AS Tyteca D . Plasma membrane lipid domains as platforms for vesicle biogenesis and shedding? Biomolecules. (2018) 8:94. doi: 10.3390/biom8030094, PMID: 30223513 PMC6164003

[44] Chen Z Han F Du Y Shi H Zhou W . Hypoxic microenvironment in cancer: molecular mechanisms and therapeutic interventions. Signal Transduct Target Ther. (2023) 8:70. doi: 10.1038/s41392-023-01332-8, PMID: 36797231 PMC9935926

[45] Lai X Zhong J Zhang B Zhu T Liao R . Exosomal non-coding RNAs: novel regulators of macrophage-linked intercellular communication in lung cancer and inflammatory lung diseases. Biomolecules. (2023) 13:536. doi: 10.3390/biom13030536, PMID: 36979471 PMC10046066

[46] To KKW Cho WCS . Exosome secretion from hypoxic cancer cells reshapes the tumor microenvironment and mediates drug resistance. Cancer Drug Resist. (2022) 5:577–94. doi: 10.20517/cdr.2022.38, PMID: 36176760 PMC9511811

[47] Li X Liu Y Zheng S Zhang T Wu J Sun Y . Role of exosomes in the immune microenvironment of ovarian cancer. Oncol Lett. (2021) 21:377. doi: 10.3892/ol.2021.12638, PMID: 33777201 PMC7988709

[48] Munir MT Kay MK Kang MH Rahman MM Al-Harrasi A Choudhury M . Tumor-associated macrophages as multifaceted regulators of breast tumor growth. Int J Mol Sci. (2021) 22:6526. doi: 10.3390/ijms22126526, PMID: 34207035 PMC8233875

[49] Othman N Jamal R Abu N . Cancer-derived exosomes as effectors of key inflammation-related players. Front Immunol. (2019) 10:2103. doi: 10.3389/fimmu.2019.02103, PMID: 31555295 PMC6737008

[50] Padoan A Plebani M Basso D . Inflammation and pancreatic cancer: focus on metabolism, cytokines, and immunity. Int J Mol Sci. (2019) 20:676. doi: 10.3390/ijms20030676, PMID: 30764482 PMC6387440

[51] Nowak M Klink M . The role of tumor-associated macrophages in the progression and chemoresistance of ovarian cancer. Cells. (2020) 9:1299. doi: 10.3390/cells9051299, PMID: 32456078 PMC7290435

[52] Salimi L Akbari A Jabbari N Mojarad B Vahhabi A Szafert S . Synergies in exosomes and autophagy pathways for cellular homeostasis and metastasis of tumor cells. Cell Biosci. (2020) 10:64. doi: 10.1186/s13578-020-00426-y, PMID: 32426106 PMC7218515

[53] Huang R Kang T Chen S . The role of tumor-associated macrophages in tumor immune evasion. J Cancer Res Clin Oncol. (2024) 150:238. doi: 10.1007/s00432-024-05777-4, PMID: 38713256 PMC11076352

[54] Wang J Long R Han Y . The role of exosomes in the tumour microenvironment on macrophage polarisation. Biochim Biophys Acta Rev Cancer. (2022) 1877:188811. doi: 10.1016/j.bbcan.2022.188811, PMID: 36208648

[55] Nogueras Pérez R Heredia-Nicolás N De Lara-Peña L López De Andrés J Marchal JA Jiménez G . Unraveling the potential of miRNAs from CSCs as an emerging clinical tool for breast cancer diagnosis and prognosis. Int J Mol Sci. (2023) 24:16010. doi: 10.3390/ijms242116010., PMID: 37958993 PMC10647353

[56] Yin Z Ma T Huang B Lin L Zhou Y Yan J . Macrophage-derived exosomal microRNA-501-3p promotes progression of pancreatic ductal adenocarcinoma through the TGFBR3-mediated TGF-β signaling pathway. J Exp Clin Cancer Res. (2019) 38:310. doi: 10.1186/s13046-019-1313-x, PMID: 31307515 PMC6631643

[57] Li X Tang M . Exosomes released from M2 macrophages transfer miR-221-3p contributed to EOC progression through targeting CDKN1B. Cancer Med. (2020) 9:5976–88. doi: 10.1002/cam4.3252, PMID: 32590883 PMC7433826

[58] Zhou J Li X Wu X Zhang T Zhu Q Wang X . Exosomes released from tumor-associated macrophages transfer miRNAs that induce a treg/th17 cell imbalance in epithelial ovarian cancer. Cancer Immunol Res. (2018) 6:1578–92. doi: 10.1158/2326-6066.CIR-17-0479, PMID: 30396909

[59] Xu S Xu H Wang W Li S Li H Li T . The role of collagen in cancer: from bench to bedside. J Transl Med. (2019) 17:309. doi: 10.1186/s12967-019-2058-1, PMID: 31521169 PMC6744664

[60] Cheng YQ Wang SB Liu JH Jin L Liu Y Li CY . Modifying the tumour microenvironment and reverting tumour cells: New strategies for treating Malignant tumours. Cell Prolif. (2020) 53:e12865. doi: 10.1111/cpr.12865, PMID: 32588948 PMC7445401

[61] Chitty JL Filipe EC Lucas MC Herrmann D Cox TR Timpson P . Recent advances in understanding the complexities of metastasis. F1000Res. (2018) 7:169. doi: 10.12688/f1000research.15064.1, PMID: 30135716 PMC6073095

[62] Mi X Xu R Hong S Xu T Zhang W Liu M . M2 macrophage-derived exosomal lncRNA AFAP1-AS1 and microRNA-26a affect cell migration and metastasis in esophageal cancer. Mol Ther Nucleic Acids. (2020) 22:779–90. doi: 10.1016/j.omtn.2020.09.035, PMID: 33230475 PMC7595846

[63] Lan J Sun L Xu F Liu L Hu F Song D . M2 macrophage-derived exosomes promote cell migration and invasion in colon cancer. Cancer Res. (2019) 79:146–58. doi: 10.1158/0008-5472.CAN-18-0014, PMID: 30401711

[64] Liang Y Liang Q Qiao L Xiao F . MicroRNAs modulate drug resistance-related mechanisms in hepatocellular carcinoma. Front Oncol. (2020) 10:920. doi: 10.3389/fonc.2020.00920, PMID: 32695666 PMC7338562

[65] Xu Z Chen Y Ma L Chen Y Liu J Guo Y . Role of exosomal non-coding RNAs from tumor cells and tumor-associated macrophages in the tumor microenvironment. Mol Ther. (2022) 30:3133–54. doi: 10.1016/j.ymthe.2022.01.046, PMID: 35405312 PMC9552915

[66] Hu Y Li D Wu A Qiu X Di W Huang L . TWEAK-stimulated macrophages inhibit metastasis of epithelial ovarian cancer via exosomal shuttling of microRNA. Cancer Lett. (2017) 393:60–7. doi: 10.1016/j.canlet.2017.02.009, PMID: 28216373

[67] Duan S Yu S Yuan T Yao S Zhang L . Corrigendum: Exogenous let-7a-5p induces A549 lung cancer cell death through BCL2L1-mediated PI3Kγ signaling pathway. Front Oncol. (2024) 14:1513956. doi: 10.3389/fonc.2024.1513956, PMID: 39687894 PMC11648416

[68] Zhang Y Tang S Gao Y Lu Z Yang Y Chen J . Application of exosomal miRNA mediated macrophage polarization in colorectal cancer: Current progress and challenges. Oncol Res. (2023) 32:61–71. doi: 10.32604/or.2023.043481, PMID: 38188683 PMC10767244

[69] Maacha S Bhat AA Jimenez L Raza A Haris M Uddin S . Extracellular vesicles-mediated intercellular communication: roles in the tumor microenvironment and anti-cancer drug resistance. Mol Cancer. (2019) 18:55. doi: 10.1186/s12943-019-0965-7, PMID: 30925923 PMC6441157

[70] Bolzoni M Toscani D Storti P Marchica V Costa F Giuliani N . Possible targets to treat myeloma-related osteoclastogenesis. Expert Rev Hematol. (2018) 11:325–36. doi: 10.1080/17474086.2018.1447921, PMID: 29495905

[71] Chistiakov DA Orekhov AN Bobryshev YV . Extracellular vesicles and atherosclerotic disease. Cell Mol Life Sci. (2015) 72:2697–708. doi: 10.1007/s00018-015-1906-2, PMID: 25894694 PMC11113133

[72] Ali Syeda Z Langden SSS Munkhzul C Lee M Song SJ . Regulatory mechanism of microRNA expression in cancer. Int J Mol Sci. (2020) 21:1723. doi: 10.3390/ijms21051723, PMID: 32138313 PMC7084905

[73] Entezari M Sadrkhanloo M Rashidi M Asnaf SE Taheriazam A Hashemi M . Non-coding RNAs and macrophage interaction in tumor progression. Crit Rev Oncol Hematol. (2022) 173:103680. doi: 10.1016/j.critrevonc.2022.103680, PMID: 35405273

[74] Wang X Xie N Zhang H Zhou W Lei J . Isoorientin ameliorates macrophage pyroptosis and atherogenesis by reducing KDM4A levels and promoting SKP1-cullin1-F-box E3 ligase-mediated NLRP3 ubiquitination. Inflammation. (2025) 48(5):3629–3648. doi: 10.1007/s10753-025-02289-2, PMID: 40133580 PMC12596395

[75] Herrington CS Poulsom R Coates PJ . Recent advances in pathology: the 2020 annual review issue of the journal of pathology. J Pathol. (2020) 250:475–9. doi: 10.1002/path.5425, PMID: 32346919

[76] Hashemi M Khosroshahi EM Chegini MK Abedi M Matinahmadi A Hosnarody YSD . miRNAs and exosomal miRNAs in lung cancer: New emerging players in tumor progression and therapy response. Pathol Res Pract. (2023) 251:154906. doi: 10.1016/j.prp.2023.154906, PMID: 37939448

[77] Wani S Man Law IK Pothoulakis C . Role and mechanisms of exosomal miRNAs in IBD pathophysiology. Am J Physiol Gastrointest Liver Physiol. (2020) 319:G646–g654. doi: 10.1152/ajpgi.00295.2020, PMID: 33026230 PMC7792667

[78] Jia Y Wei Y . Modulators of microRNA function in the immune system. Int J Mol Sci. (2020) 21:2357. doi: 10.3390/ijms21072357, PMID: 32235299 PMC7177468

[79] Jiang H Zhao H Zhang M He Y Li X Xu Y . Hypoxia induced changes of exosome cargo and subsequent biological effects. Front Immunol. (2022) 13:824188. doi: 10.3389/fimmu.2022.824188, PMID: 35444652 PMC9013908

[80] Boon RA Vickers KC . Intercellular transport of microRNAs. Arterioscler Thromb Vasc Biol. (2013) 33:186–92. doi: 10.1161/ATVBAHA.112.300139, PMID: 23325475 PMC3580056

[81] Wu T Zhao Y Zhang X Wang Y Chen Q Zhang M . Short-chain acyl post-translational modifications in cancers: Mechanisms, roles, and therapeutic implications. Cancer Commun (Lond). (2025) 45:1247–84. doi: 10.1002/cac2.70048, PMID: 40703012 PMC12531430

[82] Zhang S Chen W Zhou J Liang Q Zhang Y Su M . The benefits and safety of monoclonal antibodies: implications for cancer immunotherapy. J Inflammation Res. (2025) 18:4335–57. doi: 10.2147/JIR.S499403, PMID: 40162076 PMC11952073

[83] Khan MI Alsayed R Choudhry H Ahmad A . Exosome-mediated response to cancer therapy: modulation of epigenetic machinery. Int J Mol Sci. (2022) 23:6222. doi: 10.3390/ijms23116222, PMID: 35682901 PMC9181065

[84] Kovaleva O Sorokin M Egorova A Petrenko A Shelekhova K Gratchev A . Macrophage - tumor cell interaction beyond cytokines. Front Oncol. (2023) 13:1078029. doi: 10.3389/fonc.2023.1078029, PMID: 36910627 PMC9995642

[85] Wang L Yu Q Xiao J Chen Q Fang M Zhao H . Cigarette Smoke Extract-Treated Mouse Airway Epithelial Cells-Derived Exosomal LncRNA MEG3 Promotes M1 Macrophage Polarization and Pyroptosis in Chronic Obstructive Pulmonary Disease by Upregulating TREM-1 via m(6)A Methylation. Immune Netw. (2024) 24:e3. doi: 10.4110/in.2024.24.e3, PMID: 38725674 PMC11076299

[86] Lathigara D Kaushal D Wilson RB . Molecular mechanisms of western diet-induced obesity and obesity-related carcinogenesis-A narrative review. Metabolites. (2023) 13:675. doi: 10.3390/metabo13050675, PMID: 37233716 PMC10222258

[87] Jiang C Zhang J Wang W Shan Z Sun F Tan Y . Extracellular vesicles in gastric cancer: role of exosomal lncRNA and microRNA as diagnostic and therapeutic targets. Front Physiol. (2023) 14:1158839. doi: 10.3389/fphys.2023.1158839, PMID: 37664422 PMC10469264

[88] Momen-Heravi F Bala S . miRNA regulation of innate immunity. J Leukoc Biol. (2018). doi: 10.1002/JLB.3MIR1117-459R, PMID: 29656417

[89] Lone SN Bhat AA Wani NA Karedath T Hashem S Nisar S . miRNAs as novel immunoregulators in cancer. Semin Cell Dev Biol. (2022) 124:3–14. doi: 10.1016/j.semcdb.2021.04.013, PMID: 33926791

[90] Nail HM Chiu CC Leung CH Ahmed MMM Wang HD . Exosomal miRNA-mediated intercellular communications and immunomodulatory effects in tumor microenvironments. J BioMed Sci. (2023) 30:69. doi: 10.1186/s12929-023-00964-w, PMID: 37605155 PMC10440907

[91] İlhan A Golestani S Shafagh SG Asadi F Daneshdoust D Al-Naqeeb BZT . The dual role of microRNA (miR)-20b in cancers: Friend or foe? Cell Communication Signaling. (2023) 21:26. doi: 10.1186/s12964-022-01019-7, PMID: 36717861 PMC9885628

[92] Zhu X Shen H Yin X Yang M Wei H Chen Q . Macrophages derived exosomes deliver miR-223 to epithelial ovarian cancer cells to elicit a chemoresistant phenotype. J Exp Clin Cancer Res. (2019) 38:81. doi: 10.1186/s13046-019-1095-1, PMID: 30770776 PMC6377760

[93] Zheng P Chen L Yuan X Luo Q Liu Y Xie G . Exosomal transfer of tumor-associated macrophage-derived miR-21 confers cisplatin resistance in gastric cancer cells. J Exp Clin Cancer Res. (2017) 36:53. doi: 10.1186/s13046-017-0528-y, PMID: 28407783 PMC5390430

[94] Binenbaum Y Fridman E Yaari Z Milman N Schroeder A Ben David G . Transfer of miRNA in macrophage-derived exosomes induces drug resistance in pancreatic adenocarcinoma. Cancer Res. (2018) 78:5287–99. doi: 10.1158/0008-5472.CAN-18-0124, PMID: 30042153

[95] Li X Chen Z Ni Y Bian C Huang J Chen L . Tumor-associated macrophages secret exosomal miR-155 and miR-196a-5p to promote metastasis of non-small-cell lung cancer. Transl Lung Cancer Res. (2021) 10:1338–54. doi: 10.21037/tlcr-20-1255, PMID: 33889514 PMC8044469

[96] Ma YS Wu TM Ling CC Yu F Zhang J Cao PS . M2 macrophage-derived exosomal microRNA-155-5p promotes the immune escape of colon cancer by downregulating ZC3H12B. Mol Ther Oncolytics. (2021) 20:484–98. doi: 10.1016/j.omto.2021.02.005, PMID: 33718596 PMC7932913

[97] An B Shin CH Kwon JW Tran NL Kim AH Jeong H . M1 macrophage-derived exosomal microRNA-29c-3p suppresses aggressiveness of melanoma cells via ENPP2. Cancer Cell Int. (2024) 24:325. doi: 10.1186/s12935-024-03512-0, PMID: 39342305 PMC11438108

[98] Rudin CM Brambilla E Faivre-Finn C Sage J . Small-cell lung cancer. Nat Rev Dis Primers. (2021) 7:3. doi: 10.1038/s41572-020-00235-0, PMID: 33446664 PMC8177722

[99] Dela Cruz CS Tanoue LT Matthay RA . Lung cancer: epidemiology, etiology, and prevention. Clin Chest Med. (2011) 32:605–44. doi: 10.1016/j.ccm.2011.09.001, PMID: 22054876 PMC3864624

[100] Nazri HM Greaves E Quenby S Dragovic R Tapmeier TT Becker CM . The role of small extracellular vesicle-miRNAs in endometriosis. Hum Reprod. (2023) 38:2296–311. doi: 10.1093/humrep/dead216, PMID: 37877421 PMC10694411

[101] Thandra KC Barsouk A Saginala K Aluru JS Barsouk A . Epidemiology of lung cancer. Contemp Oncol (Pozn). (2021) 25:45–52. doi: 10.5114/wo.2021.103829, PMID: 33911981 PMC8063897

[102] Takeda Y Kobayashi S Kitakaze M Yamada D Akita H Asai A . Immuno-surgical management of pancreatic cancer with analysis of cancer exosomes. Cells. (2020) 9:1645. doi: 10.3390/cells9071645, PMID: 32659892 PMC7408222

[103] Saleh RO Hjazi A Bansal P Ahmad I Kaur H Ali SHJ . Mysterious interactions between macrophage-derived exosomes and tumors; what do we know? Pathol Res Pract. (2024) 256:155261. doi: 10.1016/j.prp.2024.155261, PMID: 38518733

[104] Yu W Liang X Li X Zhang Y Sun Z Liu Y . MicroRNA-195: a review of its role in cancers. Onco Targets Ther. (2018) 11:7109–23. doi: 10.2147/OTT.S183600, PMID: 30410367 PMC6200091

[105] Thapa R Afzal O Bhat AA Goyal A Alfawaz Altamimi AS Almalki WH . New horizons in lung cancer management through ATR/CHK1 pathway modulation. Future medicinal Chem. (2023) 15:1807–18. doi: 10.4155/fmc-2023-0164, PMID: 37877252

[106] Yang C Kim HS Song G Lim W . The potential role of exosomes derived from ovarian cancer cells for diagnostic and therapeutic approaches. J Cell Physiol. (2019) 234:21493–503. doi: 10.1002/jcp.28905, PMID: 31144314

[107] Yoshida K Yokoi A Kato T Ochiya T Yamamoto Y . The clinical impact of intra- and extracellular miRNAs in ovarian cancer. Cancer Sci. (2020) 111:3435–44. doi: 10.1111/cas.14599, PMID: 32750177 PMC7541008

[108] Pekarek L Torres-Carranza D Fraile-Martinez O García-Montero C Pekarek T Saez MA . An overview of the role of microRNAs on carcinogenesis: A focus on cell cycle, angiogenesis and metastasis. Int J Mol Sci. (2023) 24:7268. doi: 10.3390/ijms24087268, PMID: 37108432 PMC10139430

[109] Zeng Y Fu BM . Resistance mechanisms of anti-angiogenic therapy and exosomes-mediated revascularization in cancer. Front Cell Dev Biol. (2020) 8:610661. doi: 10.3389/fcell.2020.610661, PMID: 33363174 PMC7755714

[110] Song Y Kelava L Kiss I . MiRNAs in lung adenocarcinoma: role, diagnosis, prognosis, and therapy. Int J Mol Sci. (2023) 24:13302. doi: 10.3390/ijms241713302, PMID: 37686110 PMC10487838

[111] Duan S Yu S Yuan T Yao S Zhang L . Exogenous let-7a-5p induces A549 lung cancer cell death through BCL2L1-mediated PI3Kγ Signaling pathway. Front Oncol. (2019) 9:808. doi: 10.3389/fonc.2019.00808, PMID: 31508368 PMC6716507

[112] Ahmad A . Epigenetic regulation of immunosuppressive tumor-associated macrophages through dysregulated microRNAs. Semin Cell Dev Biol. (2022) 124:26–33. doi: 10.1016/j.semcdb.2021.09.001, PMID: 34556420

[113] Ashrafizadeh M Kumar AP Aref AR Zarrabi A Mostafavi E . Exosomes as promising nanostructures in diabetes mellitus: from insulin sensitivity to ameliorating diabetic complications. Int J Nanomedicine. (2022) 17:1229–53. doi: 10.2147/IJN.S350250, PMID: 35340823 PMC8943613

[114] Chen C Liu JM Luo YP . MicroRNAs in tumor immunity: functional regulation in tumor-associated macrophages. J Zhejiang Univ Sci B. (2020) 21:12–28. doi: 10.1631/jzus.B1900452, PMID: 31898439 PMC6964996

[115] Mercogliano MF Bruni S Mauro F Elizalde PV Schillaci R . Harnessing tumor necrosis factor alpha to achieve effective cancer immunotherapy. Cancers (Basel). (2021) 13:564. doi: 10.3390/cancers13030564, PMID: 33540543 PMC7985780

[116] Jiao Y Zhang T Zhang C Ji H Tong X Xia R . Exosomal miR-30d-5p of neutrophils induces M1 macrophage polarization and primes macrophage pyroptosis in sepsis-related acute lung injury. Crit Care. (2021) 25:356. doi: 10.1186/s13054-021-03775-3, PMID: 34641966 PMC8507252

[117] Metcalf G . MicroRNAs: circulating biomarkers for the early detection of imperceptible cancers via biosensor and machine-learning advances. Oncogene. (2024) 43:2135–42. doi: 10.1038/s41388-024-03076-3, PMID: 38839942 PMC11226400

[118] Cazzoli R Buttitta F Di Nicola M Malatesta S Marchetti A Rom WN . microRNAs derived from circulating exosomes as noninvasive biomarkers for screening and diagnosing lung cancer. J Thorac Oncol. (2013) 8:1156–62. doi: 10.1097/JTO.0b013e318299ac32, PMID: 23945385 PMC4123222

[119] Munagala R Aqil F Gupta RC . Exosomal miRNAs as biomarkers of recurrent lung cancer. Tumor Biol. (2016) 37:10703–14. doi: 10.1007/s13277-016-4939-8, PMID: 26867772

[120] Wang C Wang X Zhang D Sun X Wu Y Wang J . The macrophage polarization by miRNAs and its potential role in the treatment of tumor and inflammation (Review). Oncol Rep. (2023) 50:190. doi: 10.3892/or.2023.8627, PMID: 37711048 PMC10523439

[121] Bazi A Rashidi-Juybari FZ Hosseini SM Khazaee-Nasirabadi MH Ghorbani Biregani K Peymaninezhad F . Extracellular vesicle–derived microRNAs: A deep review of the latest literature investigating their role in drug resistance, prognosis, and microenvironment interactions in hematologic Malignancies. Eur J Cancer Care. (2025) 2025:5512907. doi: 10.1155/ecc/5512907

[122] Li X Xiang J Wang J Li J Wu FX Li M . FUNMarker: fusion network-based method to identify prognostic and heterogeneous breast cancer biomarkers. IEEE/ACM Trans Comput Biol Bioinform. (2021) 18:2483–91. doi: 10.1109/TCBB.2020.2973148, PMID: 32070993

[123] Feng Y Spezia M Huang S Yuan C Zeng Z Zhang L . Breast cancer development and progression: Risk factors, cancer stem cells, signaling pathways, genomics, and molecular pathogenesis. Genes Dis. (2018) 5:77–106. doi: 10.1016/j.gendis.2018.05.001, PMID: 30258937 PMC6147049

[124] Syed SN Frank AC Raue R Brüne B . MicroRNA-A tumor trojan horse for tumor-associated macrophages. Cells. (2019) 8:1482. doi: 10.3390/cells8121482, PMID: 31766495 PMC6953083

[125] Ginsburg O Yip CH Brooks A Cabanes A Caleffi M Dunstan Yataco JA . Breast cancer early detection: A phased approach to implementation. Cancer. (2020) 126 Suppl 10:2379–93. doi: 10.1002/cncr.32887, PMID: 32348566 PMC7237065

[126] Xu WX Wang DD Zhao ZQ Zhang HD Yang SJ Zhang Q . Exosomal microRNAs shuttling between tumor cells and macrophages: cellular interactions and novel therapeutic strategies. Cancer Cell Int. (2022) 22:190. doi: 10.1186/s12935-022-02594-y, PMID: 35578228 PMC9109313

[127] Łukasiewicz S Czeczelewski M Forma A Baj J Sitarz R Stanisławek A . Breast cancer-epidemiology, risk factors, classification, prognostic markers, and current treatment strategies-an updated review. Cancers (Basel). (2021) 13:4287. doi: 10.3390/cancers13174287, PMID: 34503097 PMC8428369

[128] Santos P Almeida F . Role of exosomal miRNAs and the tumor microenvironment in drug resistance. Cells. (2020) 9:1450. doi: 10.3390/cells9061450, PMID: 32545155 PMC7349227

[129] Syed SN Brüne B . Exosomal and non-exosomal microRNAs: new kids on the block for cancer therapy. Int J Mol Sci. (2022) 23:4493. doi: 10.3390/ijms23094493, PMID: 35562884 PMC9104172

[130] Muñoz JP Pérez-Moreno P Pérez Y Calaf GM . The role of microRNAs in breast cancer and the challenges of their clinical application. Diagnostics (Basel). (2023) 13:3072. doi: 10.3390/diagnostics13193072, PMID: 37835815 PMC10572677

[131] Baylie T Kasaw M Getinet M Getie G Jemal M Nigatu A . The role of miRNAs as biomarkers in breast cancer. Front Oncol. (2024) 14:1374821. doi: 10.3389/fonc.2024.1374821, PMID: 38812786 PMC11133523

[132] Strizova Z Benesova I Bartolini R Novysedlak R Cecrdlova E Foley LK . M1/M2 macrophages and their overlaps - myth or reality? Clin Sci (Lond). (2023) 137:1067–93. doi: 10.1042/CS20220531, PMID: 37530555 PMC10407193

[133] Clements ME Johnson RW . Breast cancer dormancy in bone. Curr Osteoporos Rep. (2019) 17:353–61. doi: 10.1007/s11914-019-00532-y, PMID: 31468498 PMC6819229

[134] Walker ND Elias M Guiro K Bhatia R Greco SJ Bryan M . Exosomes from differentially activated macrophages influence dormancy or resurgence of breast cancer cells within bone marrow stroma. Cell Death Dis. (2019) 10:59. doi: 10.1038/s41419-019-1304-z, PMID: 30683851 PMC6347644

[135] Neophytou CM Panagi M Stylianopoulos T Papageorgis P . The role of tumor microenvironment in cancer metastasis: molecular mechanisms and therapeutic opportunities. Cancers (Basel). (2021) 13:2053. doi: 10.3390/cancers13092053, PMID: 33922795 PMC8122975

[136] Liu J Ren L Li S Li W Zheng X Yang Y . The biology, function, and applications of exosomes in cancer. Acta Pharm Sin B. (2021) 11:2783–97. doi: 10.1016/j.apsb.2021.01.001, PMID: 34589397 PMC8463268

[137] Jin Y Xing J Xu K Liu D Zhuo Y . Exosomes in the tumor microenvironment: Promoting cancer progression. Front Immunol. (2022) 13:1025218. doi: 10.3389/fimmu.2022.1025218, PMID: 36275738 PMC9584056

[138] Yang M Chen J Su F Yu B Su F Lin L . Microvesicles secreted by macrophages shuttle invasion-potentiating microRNAs into breast cancer cells. Mol Cancer. (2011) 10:117. doi: 10.1186/1476-4598-10-117, PMID: 21939504 PMC3190352

[139] Moradi-Chaleshtori M Hashemi SM Soudi S Bandehpour M Mohammadi-Yeganeh S . Tumor-derived exosomal microRNAs and proteins as modulators of macrophage function. J Cell Physiol. (2019) 234:7970–82. doi: 10.1002/jcp.27552, PMID: 30378104

[140] Huang X Cao J Zu X . Tumor-associated macrophages: An important player in breast cancer progression. Thorac Cancer. (2022) 13:269–76. doi: 10.1111/1759-7714.14268, PMID: 34914196 PMC8807249

[141] Allison E Edirimanne S Matthews J Fuller SJ . Breast cancer survival outcomes and tumor-associated macrophage markers: A systematic review and meta-analysis. Oncol Ther. (2023) 11:27–48. doi: 10.1007/s40487-022-00214-3, PMID: 36484945 PMC9935786

[142] Li Y Zhao L Shi B Ma S Xu Z Ge Y . Functions of miR-146a and miR-222 in Tumor-associated Macrophages in Breast Cancer. Sci Rep. (2015) 5:18648. doi: 10.1038/srep18648, PMID: 26689540 PMC4686897

[143] Zare E Yaghoubi SM Khoshnazar M Jafari Dargahlou S Machhar JS Zheng Z . MicroRNAs in cancer immunology: master regulators of the tumor microenvironment and immune evasion, with therapeutic potential. Cancers (Basel). (2025) 17:2172. doi: 10.3390/cancers17132172, PMID: 40647470 PMC12248500

[144] Jelovac D Armstrong DK . Recent progress in the diagnosis and treatment of ovarian cancer. CA Cancer J Clin. (2011) 61:183–203. doi: 10.3322/caac.20113, PMID: 21521830 PMC3576854

[145] Mohan A Agarwal S Clauss M Britt NS Dhillon NK . Extracellular vesicles: novel communicators in lung diseases. Respir Res. (2020) 21:175. doi: 10.1186/s12931-020-01423-y, PMID: 32641036 PMC7341477

[146] Konstantinopoulos PA Norquist B Lacchetti C Armstrong D Grisham RN Goodfellow PJ . Germline and somatic tumor testing in epithelial ovarian cancer: ASCO guideline. J Clin Oncol. (2020) 38:1222–45. doi: 10.1200/JCO.19.02960, PMID: 31986064 PMC8842911

[147] Malla RR Kiran P . Tumor microenvironment pathways: Cross regulation in breast cancer metastasis. Genes Dis. (2022) 9:310–24. doi: 10.1016/j.gendis.2020.11.015, PMID: 35224148 PMC8843880

[148] Matsuzaka Y Yashiro R . Regulation of extracellular vesicle-mediated immune responses against antigen-specific presentation. Vaccines (Basel). (2022) 10:1691. doi: 10.3390/vaccines10101691, PMID: 36298556 PMC9607341

[149] Momenimovahed Z Tiznobaik A Taheri S Salehiniya H . Ovarian cancer in the world: epidemiology and risk factors. Int J Womens Health. (2019) 11:287–99. doi: 10.2147/IJWH.S197604, PMID: 31118829 PMC6500433

[150] Ali AT Al-Ani O Al-Ani F . Epidemiology and risk factors for ovarian cancer. Prz Menopauzalny. (2023) 22:93–104. doi: 10.5114/pm.2023.128661, PMID: 37674925 PMC10477765

[151] Kotsopoulos J Lubinski J Gronwald J Cybulski C Demsky R Neuhausen SL . Factors influencing ovulation and the risk of ovarian cancer in BRCA1 and BRCA2 mutation carriers. Int J Cancer. (2015) 137:1136–46. doi: 10.1002/ijc.29386, PMID: 25482078 PMC4458227

[152] Li Y Jin D Xie W Wen L Chen W Xu J . Mesenchymal stem cells-derived exosomes: A possible therapeutic strategy for osteoporosis. Curr Stem Cell Res Ther. (2018) 13:362–8. doi: 10.2174/1574888X13666180403163456, PMID: 29623851

[153] Fasoulakis Z Psarommati MZ Papapanagiotou A Pergialiotis V Koutras A Douligeris A . MicroRNAs can influence ovarian cancer progression by dysregulating integrin activity. Cancers (Basel). (2023) 15:4449. doi: 10.3390/cancers15184449, PMID: 37760437 PMC10526761

[154] Chou J Provot S Werb Z . GATA3 in development and cancer differentiation: cells GATA have it! J Cell Physiol. (2010) 222:42–9. doi: 10.1002/jcp.21943, PMID: 19798694 PMC2915440

[155] Ben Hamouda S Essafi-Benkhadir K . Interplay between signaling pathways and tumor microenvironment components: A paradoxical role in colorectal cancer. Int J Mol Sci. (2023) 24:5600. doi: 10.3390/ijms24065600, PMID: 36982677 PMC10057671

[156] El-Arabey AA Denizli M Kanlikilicer P Bayraktar R Ivan C Rashed M . GATA3 as a master regulator for interactions of tumor-associated macrophages with high-grade serous ovarian carcinoma. Cell Signalling. (2020) 68:109539. doi: 10.1016/j.cellsig.2020.109539, PMID: 31935430

[157] Khatun M Ray RB . Mechanisms underlying hepatitis C virus-associated hepatic fibrosis. Cells. (2019) 8:1249. doi: 10.3390/cells8101249, PMID: 31615075 PMC6829586

[158] Kwon Y Kim M Kim Y Jung HS Jeoung D . Exosomal microRNAs as mediators of cellular interactions between cancer cells and macrophages. Front Immunol. (2020) 11:1167. doi: 10.3389/fimmu.2020.01167, PMID: 32595638 PMC7300210

[159] Whiteside TL . What are regulatory T cells (Treg) regulating in cancer and why? Semin Cancer Biol. (2012) 22:327–34. doi: 10.1016/j.semcancer.2012.03.004, PMID: 22465232 PMC3385925

[160] Holmberg R Robinson M Gilbert SF Lujano-Olazaba O Waters JA Kogan E . TWEAK-fn14-relB signaling cascade promotes stem cell-like features that contribute to post-chemotherapy ovarian cancer relapse. Mol Cancer Res. (2023) 21:170–86. doi: 10.1158/1541-7786.MCR-22-0486, PMID: 36214671 PMC9890141

[161] Xu C Chen J Tan M Tan Q . The role of macrophage polarization in ovarian cancer: from molecular mechanism to therapeutic potentials. Front Immunol. (2025) 16:1543096. doi: 10.3389/fimmu.2025.1543096, PMID: 40330466 PMC12052780

[162] Gallo P Silletta M Prinzi FL Farolfi T Coppola A . Hepatocellular carcinoma and non-alcoholic fatty liver disease: A modern context for an ancient disease. J Clin Med. (2023) 12:4605. doi: 10.3390/jcm12144605, PMID: 37510720 PMC10380839

[163] Chidambaranathan-Reghupaty S Fisher PB Sarkar D . Hepatocellular carcinoma (HCC): Epidemiology, etiology and molecular classification. Adv Cancer Res. (2021) 149:1–61. doi: 10.1016/bs.acr.2020.10.001, PMID: 33579421 PMC8796122

[164] El-Serag HB . Epidemiology of viral hepatitis and hepatocellular carcinoma. Gastroenterology. (2012) 142:1264–1273.e1261. doi: 10.1053/j.gastro.2011.12.061, PMID: 22537432 PMC3338949

[165] Hadad S Khalaji A Sarmadian AJ Sarmadian PJ Janagard EM Baradaran B . Tumor-associated macrophages derived exosomes; from pathogenesis to therapeutic opportunities. Int Immunopharmacol. (2024) 136:112406. doi: 10.1016/j.intimp.2024.112406, PMID: 38850795

[166] Morishita A Oura K Tadokoro T Fujita K Tani J Masaki T . MicroRNAs in the pathogenesis of hepatocellular carcinoma: A review. Cancers (Basel). (2021) 13:514. doi: 10.3390/cancers13030514, PMID: 33572780 PMC7866004

[167] Fedele M Cerchia L Chiappetta G . The epithelial-to-mesenchymal transition in breast cancer: focus on basal-like carcinomas. Cancers (Basel). (2017) 9:134. doi: 10.3390/cancers9100134, PMID: 28974015 PMC5664073

[168] Luong AB Do HQ Tarchi P Bonazza D Bottin C Cabral LKD . The mRNA distribution of cancer stem cell marker CD90/thy-1 is comparable in hepatocellular carcinoma of eastern and western populations. Cells. (2020) 9:2672. doi: 10.3390/cells9122672, PMID: 33322687 PMC7764111

[169] Wang Y Wang B Xiao S Li Y Chen Q . miR-125a/b inhibits tumor-associated macrophages mediated in cancer stem cells of hepatocellular carcinoma by targeting CD90. J Cell Biochem. (2019) 120:3046–55. doi: 10.1002/jcb.27436, PMID: 30536969

[170] Wang HC Yin WX Jiang M Han JY Kuai XW Sun R . Function and biomedical implications of exosomal microRNAs delivered by parenchymal and nonparenchymal cells in hepatocellular carcinoma. World J Gastroenterol. (2023) 29:5435–51. doi: 10.3748/wjg.v29.i39.5435, PMID: 37900996 PMC10600808

[171] Aucher A Rudnicka D Davis DM . MicroRNAs transfer from human macrophages to hepato-carcinoma cells and inhibit proliferation. J Immunol. (2013) 191:6250–60. doi: 10.4049/jimmunol.1301728, PMID: 24227773 PMC3858238

[172] Singal AK Bataller R Ahn J Kamath PS Shah VH . ACG clinical guideline: alcoholic liver disease. Am J Gastroenterol. (2018) 113:175–94. doi: 10.1038/ajg.2017.469, PMID: 29336434 PMC6524956

[173] Babuta M Furi I Bala S Bukong TN Lowe P Catalano D . Dysregulated autophagy and lysosome function are linked to exosome production by micro-RNA 155 in alcoholic liver disease. Hepatology. (2019) 70:2123–41. doi: 10.1002/hep.30766, PMID: 31090940 PMC7453183

[174] Chung KPS Leung RWH Lee TKW . Hampering stromal cells in the tumor microenvironment as a therapeutic strategy to destem cancer stem cells. Cancers (Basel). (2021) 13:3191. doi: 10.3390/cancers13133191, PMID: 34202411 PMC8268361

[175] McGuigan A Kelly P Turkington RC Jones C Coleman HG Mccain RS . Pancreatic cancer: A review of clinical diagnosis, epidemiology, treatment and outcomes. World J Gastroenterol. (2018) 24:4846–61. doi: 10.3748/wjg.v24.i43.4846, PMID: 30487695 PMC6250924

[176] Sarantis P Koustas E Papadimitropoulou A Papavassiliou AG Karamouzis MV . Pancreatic ductal adenocarcinoma: Treatment hurdles, tumor microenvironment and immunotherapy. World J Gastrointest Oncol. (2020) 12:173–81. doi: 10.4251/wjgo.v12.i2.173, PMID: 32104548 PMC7031151

[177] Ke TM Lophatananon A Muir KR . Risk factors associated with pancreatic cancer in the UK biobank cohort. Cancers (Basel). (2022) 14:4991. doi: 10.3390/cancers14204991, PMID: 36291775 PMC9599736

[178] Rawla P Barsouk A . Epidemiology of gastric cancer: global trends, risk factors and prevention. Prz Gastroenterol. (2019) 14:26–38. doi: 10.5114/pg.2018.80001, PMID: 30944675 PMC6444111

[179] Kozłowska M Śliwińska A . The link between diabetes, pancreatic tumors, and miRNAs-new players for diagnosis and therapy? Int J Mol Sci. (2023) 24:10252. doi: 10.3390/ijms241210252, PMID: 37373398 PMC10299694

[180] Tu H Costa M . XIAP’s profile in human cancer. Biomolecules. (2020) 10:1493. doi: 10.3390/biom10111493, PMID: 33138314 PMC7692959

[181] Li S Sun J Yang J Zhang L Wang L Wang X . XIAP expression is associated with pancreatic carcinoma outcome. Mol Clin Oncol. (2013) 1:305–8. doi: 10.3892/mco.2013.58, PMID: 24649165 PMC3915639

[182] Yin Z Zhou Y Ma T Chen S Shi N Zou Y . Down-regulated lncRNA SBF2-AS1 in M2 macrophage-derived exosomes elevates miR-122-5p to restrict XIAP, thereby limiting pancreatic cancer development. J Cell Mol Med. (2020) 24:5028–38. doi: 10.1111/jcmm.15125, PMID: 32301277 PMC7205800

[183] Sawicki T Ruszkowska M Danielewicz A Niedźwiedzka E Arłukowicz T Przybyłowicz KE . A review of colorectal cancer in terms of epidemiology, risk factors, development, symptoms and diagnosis. Cancers (Basel). (2021) 13:2025. doi: 10.3390/cancers13092025, PMID: 33922197 PMC8122718

[184] Nittayaboon K Leetanaporn K Sangkhathat S Roytrakul S Navakanitworakul R . Proteomic analysis of butyrate-resistant colorectal cancer-derived exosomes reveals potential resistance to anti-cancer drugs. Discov Med. (2024) 36:1306–15. doi: 10.24976/Discov.Med.202436185.121, PMID: 38926117

[185] Rawla P Sunkara T Barsouk A . Epidemiology of colorectal cancer: incidence, mortality, survival, and risk factors. Prz Gastroenterol. (2019) 14:89–103. doi: 10.5114/pg.2018.81072, PMID: 31616522 PMC6791134

[186] Ionescu VA Gheorghe G Bacalbasa N Chiotoroiu AL Diaconu C . Colorectal cancer: from risk factors to oncogenesis. Med (Kaunas). (2023) 59:1646. doi: 10.3390/medicina59091646, PMID: 37763765 PMC10537191

[187] Sado AI Batool W Ahmed A Zafar S Patel SK Mohan A . Role of microRNA in colorectal carcinoma (CRC): a narrative review. Ann Med Surg (Lond). (2024) 86:308–18. doi: 10.1097/MS9.0000000000001494, PMID: 38222721 PMC10783342

[188] Wai Hon K Zainal Abidin SA Othman I Naidu R . Insights into the Role of microRNAs in Colorectal Cancer (CRC) Metabolism. Cancers (Basel). (2020) 12:2462. doi: 10.3390/ijms241210252, PMID: 32878019 PMC7565715

[189] Xiao G Tang H Wei W Li J Ji L Ge J . Aberrant expression of microRNA-15a and microRNA-16 synergistically associates with tumor progression and prognosis in patients with colorectal cancer. Gastroenterol Res Pract. (2014) 2014:364549. doi: 10.1155/2014/364549, PMID: 25435873 PMC4236961

[190] Liebl MC Hofmann TG . The role of p53 signaling in colorectal cancer. Cancers (Basel). (2021) 13:2125. doi: 10.3390/cancers13092125, PMID: 33924934 PMC8125348

[191] Cooks T Pateras IS Jenkins LM Patel KM Robles AI Morris J . Mutant p53 cancers reprogram macrophages to tumor supporting macrophages via exosomal miR-1246. Nat Commun. (2018) 9:771. doi: 10.1038/s41467-018-03224-w, PMID: 29472616 PMC5823939

[192] Wawro M Wawro K Kochan J Solecka A Sowinska W Lichawska-Cieslar A . ZC3H12B/MCPIP2, a new active member of the ZC3H12 family. Rna. (2019) 25:840–56. doi: 10.1261/rna.071381.119, PMID: 30988100 PMC6573786

[193] Klampfer L . Cytokines, inflammation and colon cancer. Curr Cancer Drug Targets. (2011) 11:451–64. doi: 10.2174/156800911795538066, PMID: 21247378 PMC3540985

[194] Yimin E Lu C Zhu K Li W Sun J Ji P . Function and mechanism of exosomes derived from different cells as communication mediators in colorectal cancer metastasis. iScience. (2024) 27:109350. doi: 10.1016/j.isci.2024.109350, PMID: 38500820 PMC10945197

[195] Sinha D Roy S Saha P Chatterjee N Bishayee A . Trends in research on exosomes in cancer progression and anticancer therapy. Cancers (Basel). (2021) 13:326. doi: 10.3390/cancers13020326, PMID: 33477340 PMC7829710

[196] Onciul R Brehar FM Toader C Covache-Busuioc RA Glavan LA Bratu BG . Deciphering glioblastoma: fundamental and novel insights into the biology and therapeutic strategies of gliomas. Curr Issues Mol Biol. (2024) 46:2402–43. doi: 10.3390/cimb46030153, PMID: 38534769 PMC10969338

[197] Baranwal S Alahari SK . miRNA control of tumor cell invasion and metastasis. Int J Cancer. (2010) 126:1283–90. doi: 10.1002/ijc.25014, PMID: 19877123 PMC2950784

[198] Peng Y Croce CM . The role of MicroRNAs in human cancer. Signal Transduction Targeted Ther. (2016) 1:15004. doi: 10.1038/sigtrans.2015.4, PMID: 29263891 PMC5661652

[199] Sell MC Ramlogan-Steel CA Steel JC Dhungel BP . MicroRNAs in cancer metastasis: biological and therapeutic implications. Expert Rev Mol Med. (2023) 25:e14. doi: 10.1017/erm.2023.7, PMID: 36927814 PMC10407223

[200] Zheng P Luo Q Wang W Li J Wang T Wang P . Tumor-associated macrophages-derived exosomes promote the migration of gastric cancer cells by transfer of functional Apolipoprotein E. Cell Death Dis. (2018) 9:434. doi: 10.1038/s41419-018-0465-5, PMID: 29567987 PMC5864742

[201] Li Z Suo B Long G Gao Y Song J Zhang M . Exosomal miRNA-16-5p derived from M1 macrophages enhances T cell-dependent immune response by regulating PD-L1 in gastric cancer. Front Cell Dev Biol. (2020) 8:572689. doi: 10.3389/fcell.2020.572689, PMID: 33330451 PMC7734296

[202] Gao H Ma J Cheng Y Zheng P . Exosomal transfer of macrophage-derived miR-223 confers doxorubicin resistance in gastric cancer. Onco Targets Ther. (2020) 13:12169–79. doi: 10.2147/OTT.S283542, PMID: 33268995 PMC7701146

[203] Buruiană A Florian ŞI Florian AI Timiş TL Mihu CM Miclăuş M . The roles of miRNA in glioblastoma tumor cell communication: diplomatic and aggressive negotiations. Int J Mol Sci. (2020) 21:1950. doi: 10.3390/ijms21061950, PMID: 32178454 PMC7139390

[204] Ordóñez-Rubiano EG Rincón-Arias N Espinosa S Shelton WJ Salazar AF Cómbita A . The potential of miRNA-based approaches in glioblastoma: An update in current advances and future perspectives. Curr Res Pharmacol Drug Discov. (2024) 7:100193. doi: 10.1016/j.crphar.2024.100193, PMID: 39055532 PMC11268206

[205] Qian M Wang S Guo X Wang J Zhang Z Qiu W . Hypoxic glioma-derived exosomes deliver microRNA-1246 to induce M2 macrophage polarization by targeting TERF2IP via the STAT3 and NF-κB pathways. Oncogene. (2020) 39:428–42. doi: 10.1038/s41388-019-0996-y, PMID: 31485019

[206] Guo L Fu Z Li H Wei R Guo J Wang H . Smart hydrogel: A new platform for cancer therapy. Adv Colloid Interface Sci. (2025) 340:103470. doi: 10.1016/j.cis.2025.103470, PMID: 40086017

[207] Zhang M Hu S Liu L Dang P Liu Y Sun Z . Engineered exosomes from different sources for cancer-targeted therapy. Signal Transduct Target Ther. (2023) 8:124. doi: 10.1038/s41392-023-01382-y, PMID: 36922504 PMC10017761

[208] Gharavi AT Hanjani NA Movahed E Doroudian M . The role of macrophage subtypes and exosomes in immunomodulation. Cell Mol Biol Lett. (2022) 27:83. doi: 10.1186/s11658-022-00384-y, PMID: 36192691 PMC9528143

[209] Yadav A Xuan Y Sen CK Ghatak S . Standardized reporting of research on exosomes to ensure rigor and reproducibility. Adv Wound Care (New Rochelle). (2024) 13:584–99. doi: 10.1089/wound.2024.0093, PMID: 38888007 PMC12344122

[210] Nieuwland R Falcón-Pérez JM Théry C Witwer KW . Rigor and standardization of extracellular vesicle research: Paving the road towards robustness. J Extracell Vesicles. (2020) 10:e12037. doi: 10.1002/jev2.12037, PMID: 33343835 PMC7735957

[211] Cheng K Kalluri R . Guidelines for clinical translation and commercialization of extracellular vesicles and exosomes based therapeutics. Extracellular Vesicle. (2023) 2:100029. doi: 10.1016/j.vesic.2023.100029

